# HSP90 regulates temperature-dependent seedling growth in *Arabidopsis* by stabilizing the auxin co-receptor F-box protein TIR1

**DOI:** 10.1038/ncomms10269

**Published:** 2016-01-05

**Authors:** Renhou Wang, Yi Zhang, Martin Kieffer, Hong Yu, Stefan Kepinski, Mark Estelle

**Affiliations:** 1 Section of Cell and Developmental Biology, University of San Diego California, Howard Hughes Medical Institute, 9500 Gilman Dr, La Jolla, California 92093, USA; 2 Centre for Plant Sciences, Faculty of Biological Sciences, University of Leeds, Leeds LS2 9JT, UK

## Abstract

Recent studies have revealed that a mild increase in environmental temperature stimulates the growth of *Arabidopsis* seedlings by promoting biosynthesis of the plant hormone auxin. However, little is known about the role of other factors in this process. In this report, we show that increased temperature promotes rapid accumulation of the TIR1 auxin co-receptor, an effect that is dependent on the molecular chaperone HSP90. In addition, we show that HSP90 and the co-chaperone SGT1 each interact with TIR1, confirming that TIR1 is an HSP90 client. Inhibition of HSP90 activity results in degradation of TIR1 and interestingly, defects in a range of auxin-mediated growth processes at lower as well as higher temperatures. Our results indicate that HSP90 and SGT1 integrate temperature and auxin signalling in order to regulate plant growth in a changing environment.

## Abstract (web summary)

A moderate increase in temperature promotes hypocotyl elongation in *Arabidopsis*. Here, Wang *et al.* show that elevated temperature not only increases auxin biosynthesis but also acts via the co-chaperones HSP90 and SGT1 to stabilize the TIR1 auxin receptor.

## Graphical abstract

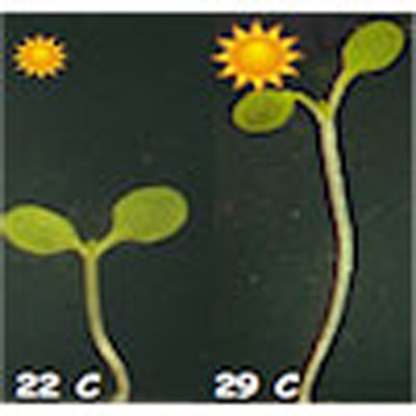

Temperature is a key environmental factor with diverse effects on plant growth and development. Extremely high temperature can severely impact growth, and plants have evolved complex mechanisms to adapt to heat stress. Extensive studies have revealed that various heat stress response factors (HSFs) and heat-shock proteins (HSPs) play central roles in heat sensing and signalling^1^,^2^,^3^,^4^. Apart from the classical heat-shock response, a mild change in ambient temperature (between 16 and 29 °C) can dramatically alter plant growth and development, and significantly affect crop yield^5^,^6^. So far, HSFs and HSPs have not been shown to play a role in these responses. In contrast, recent studies indicate that the transcription factor phytochrome-interacting factor 4 (PIF4) and the versatile plant hormone auxin play pivotal roles in ambient temperature-regulated hypocotyl growth in *Arabidopsis*^7^,^8^. At higher temperature, PIF4 acts to promote expression of several auxin biosynthetic genes resulting in increased auxin levels and hypocotyl growth. Ambient temperature signalling also appears to involve changes in chromatin structure mediated by histone H2A.Z^9^. However, the precise mechanism of thermosensing remains unclear^7^,^8^.

Auxin is perceived by the TRANSPORT INHIBITOR RESPONSE1/AUXIN RESPONSE F-box (TIR1/AFB) auxin co-receptors. These proteins are subunits of ubiquitin protein ligases called SCF^TIR1/AFB^ together with CULLIN1, *Arabidopsis* SKP1-related and RING BOX1. Auxin acts by directly binding to the TIR1/AFB proteins, and promoting the degradation of transcriptional repressors called Aux/IAA proteins^10^,^11^,^12^,^13^,^14^. The regulation of Aux/IAA degradation has been extensively studied. In contrast, there is little information on the regulation of TIR1/AFB stability. We have recently shown that TIR1 is an unstable protein in seedlings raising the possibility that changes in TIR1 stability may influence auxin signalling^15^. For example, we show that stability of the TIR1/AFB proteins is affected by their ability to assemble into an SCF complex^15^.

Assembly and regulation of SCF complexes such as SCF^TIR1^ is a highly dynamic process that involves a number of proteins and protein complexes including the ubiquitin-like protein Nedd8 (RELATED TO UBIQUITIN in *Arabidopsis*), the COP9 signalosome (CSN) complex, and the cullin-associated Nedd8-disassociated (CAND1) protein^16^,^17^,^18^,^19^,^20^,^21^. These proteins act to regulate activity of the SCF and enable the exchange of substrate adapters as required during changing cellular conditions. As the *Arabidopsis* genome encodes hundreds of FBPs, the challenge of regulating SCF assembly is particularly acute. Indeed, the RUB1, CSN and CAND1 have all been implicated in SCF^TIR1/AFB^ function^19^,^22^,^23^,^24^.

In addition to these factors, genetic studies show that a protein called SUPPRESSOR OF G2 ALLELE SKP1 (SGT1) is required for auxin response^25^. There are two *SGT1* genes in *Arabidopsis* called *SGT1a* and *SGT1b.* A mutant allele of *SGT1b,* called *enhancer of tir1 auxin resistance 3* (*eta3*), was isolated as an enhancer of the *tir1-1* mutant, implicating SGT1 in auxin response^25^. SGT1 is a co-chaperone of HEAT SHOCK FACTOR 90 (HSP90) and has been implicated in a variety of processes in eukaryotes. The protein was first identified in budding yeast and is required for assembly of kinetochores and activity of the E3 ligase SCF^Cdc4p^ (refs 26, 27). SGT1 and HSP90 play critical roles in regulating abiotic and biotic stress responses^2^,^4^. For example, they are required for normal function of nucleotide-binding domain leucine-rich repeat (NLR) immune sensor proteins in both plants and animals^28^,^29^. However, their role in ambient temperature sensing and signalling has not been investigated.

In this study, we show that the HSP90-SGT1 chaperone system is required for the plants' response to an increase in ambient temperature. TIR1 is rapidly stabilized at 29 °C, a change that is HSP90 dependent and associated with increased seedling growth. Further, we show that both HSP90 and SGT1 are in a complex with TIR1 in plants. In addition, the mutant SGT1b protein encoded by the *eta3* gene displays reduced binding to TIR1, suggesting that auxin resistance in *eta3* is directly related to TIR1 levels or function. Our data suggest that the HSP90-TIR1 module integrates environmental temperature and auxin signalling to regulate plant development and reveals a link between the molecular networks regulating plant growth response to ambient temperature and the HSPs-HSFs module that plays a central role in heat sensing and signalling.

## Results

### Temperature-dependent growth requires HSP90

**Figure 1:**
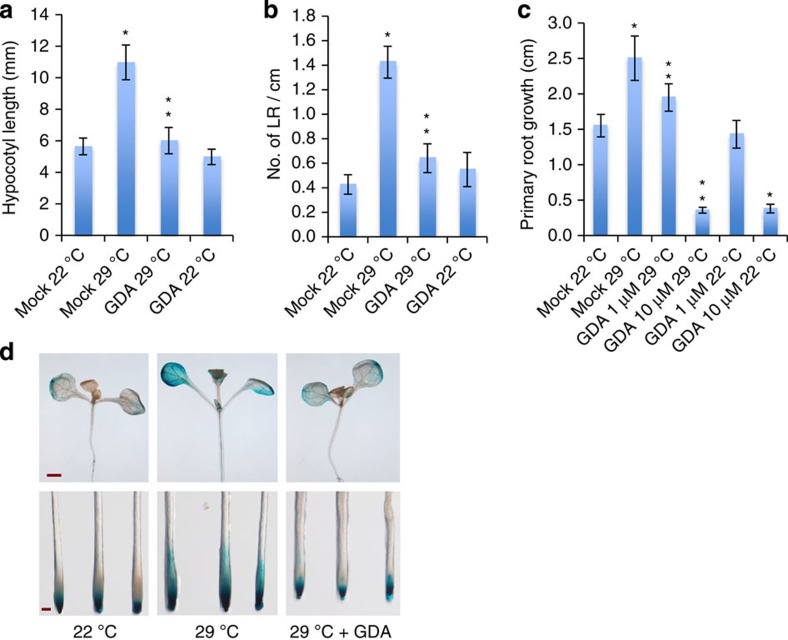
HSP90 is required for plant response to an ambient temperature increase. (**a**–**c**) GDA inhibits the positive effect of elevated temperature (29 °C) on hypocotyl elongation (**a**), number of emerged lateral roots (**b**) and primary root growth (**c**). Error bars are s.d., *n*=10–12. *Significant difference with 22 °C, **significant difference with mock (*t*-test *P*<0.01). (**d**) Five-day-old *DR5:GUS* seedlings were shifted to 29 °C and stained for GUS after 24 h. Scale bars, 1 cm(upper panels) and 200 μm (lower panels). The concentration of GDA used in **a**,**b**,**d** is 10 μM.

HSP90 has been implicated in a wide range of signalling pathways in both plants and animals^30^,^31^,^32^,^33^,^34^. In animals, HSP90 has been shown to play a fundamental role in heat-shock responses and numerous processes in non-stress conditions^33^,^34^. To determine if HSP90 has an important role in high ambient temperature promoted plant growth, we grew *Arabidopsis* seedlings at 22 and 29 °C, and treated them with the HSP90 inhibitor geldanamycin (GDA). GDA is a highly specific inhibitor of HSP90 activity without known off-target activity^30^. In this experiment, 5-day-old seedlings were transferred from 22 to 29 °C and hypocotyl length was measured after an additional 4 days in the absence and presence of GDA. As expected, hypocotyl length was stimulated by growth at the higher temperature (Fig. 1a). However, in the presence of GDA this effect was eliminated.

In our previous work, we reported that increased ambient temperature also altered lateral root formation, although we did not provide details^35^. We therefore examined the roots of seedlings shifted to 29 °C as described above. The results in Fig. 1b show that this treatment enhanced lateral root formation. Surprisingly, we found that elevated temperature also significantly promoted primary root growth (Fig. 1c). To confirm that these responses are related to auxin, we used the *tir1-1 afb2-3* double mutant, known be auxin resistant and display reduced auxin-induced lateral root formation^36^. We found that the double mutant is also affected with respect to temperature-induced growth responses indicating that the auxin receptors are required for these responses (Supplementary Fig. 1a,b). As for the hypocotyl response, we found that GDA inhibited temperature-induced changes in root growth (Fig. 1b,c). These results indicate that HSP90 is required for the effects of elevated temperature on both root and hypocotyl growth. We also tested the effects of two other structurally unrelated HSP90 inhibitors on the temperature response. Radiciol inhibits HSP90 by binding to the ATP-binding site, whereas (−)-epigallocatechin gallate binds to the C-terminus and inhibits dimerization of the protein^37^. Both inhibitors affected seedling growth in the same way as GDA (Supplementary Fig. 2a–f).

According to the model established from studies of hypocotyl growth, elevated temperature stimulates accumulation of auxin, which subsequently activates auxin responsive genes. To determine if HSP90 is required for the effect of temperature on auxin-responsive genes, we tested the effect of GDA on expression of the auxin reporter *DR5:GUS* at 22 and 29 °C. GUS levels were strongly elevated at 29 °C in both the root and shoot but as for the growth responses, this effect was strongly inhibited by GDA (Fig. 1d). We also examined three auxin responsive genes, *GH3.17, IAA19* and *IAA5*, and found that in each case, GDA prevented the characteristic positive effect of 29 °C on gene expression (Supplementary Fig. 3). Thus, our data suggest that HSP90 plays an important role in high temperature-regulated plant growth by affecting auxin-related processes. However, GDA did not affect the expression of *YUCCA8*, an auxin biosynthetic gene that is activated by high temperature (Supplementary Fig. 3)^38^. This result implies that HSP90 does not regulate the temperature growth response by modulating auxin synthesis, but rather through other processes such as auxin signalling.

### The chaperone HSP90 is required for auxin response

**Figure 2:**
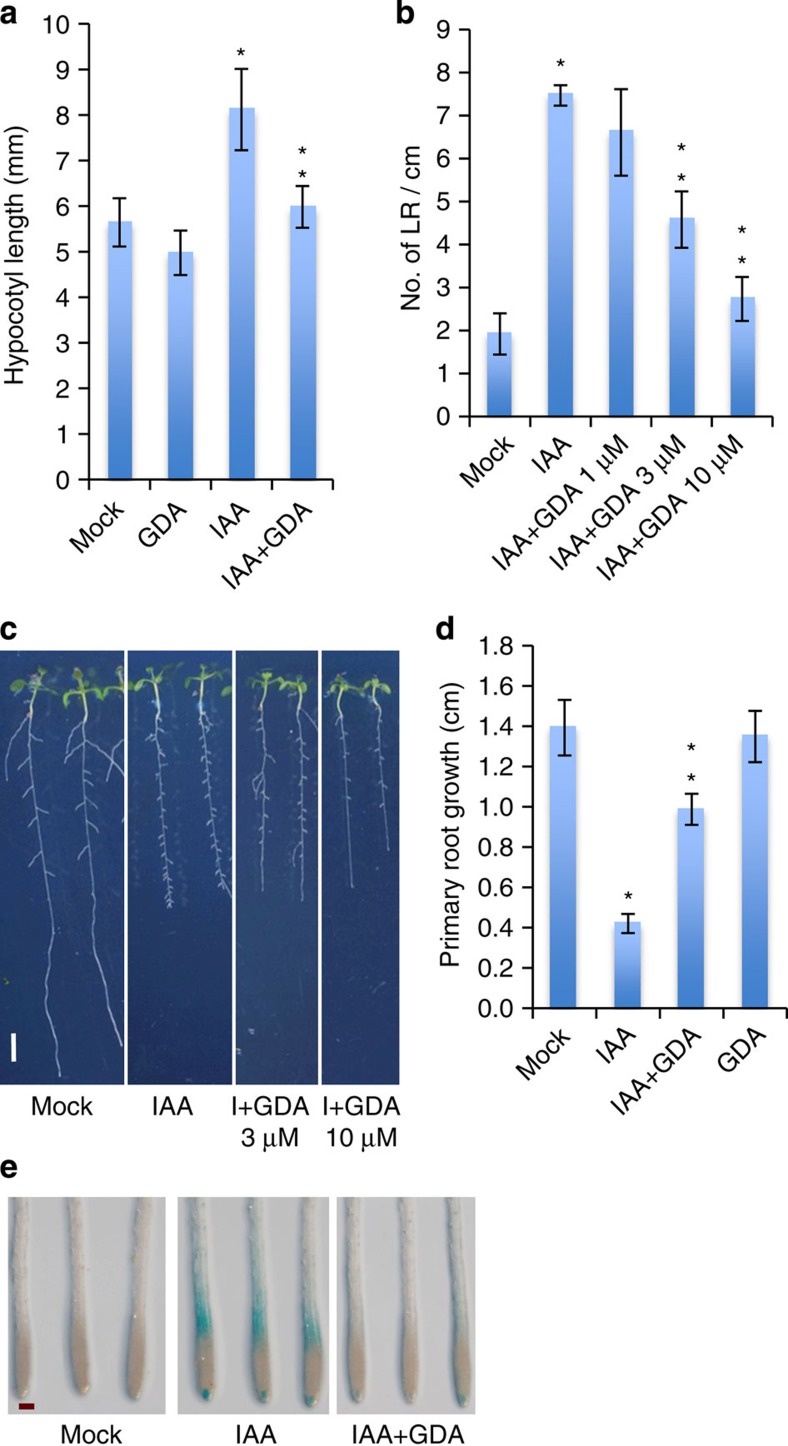
Inhibition of HSP90 activity with the specific inhibitor GDA suppresses plant responses to auxin. (**a**–**d**) Four- or five-day-old Col-0 wild-type seedlings were transferred to plates with IAA and/or GDA for various times (**a**, 3 days; **b**–**d**, 4 days). For **d**, we plotted primary root growth after transfer to IAA plates. The concentration of IAA used was: (**a**) 2 μM, (**b**,**c**) 1 μM and (**d**) 0.02 μM. The concentration of GDA used is: (**a**) 5 μM, (**b**,**c**) 1, 3 and 10 μM and (**d**) 2 μM. Error bars are s.d., *n*=10–12. *Significant difference with mock, **significant difference with IAA. (**e**) Five-day-old *DR5:GUS* seedlings were treated with IAA (2 μM) with and without GDA (10 μM) for 24 h and stained for GUS. Scale bars, 5 cm (**c**) and 200 μm (**e**).

The results described above suggest that HSP90 affects temperature-promoted growth by regulating auxin response. This is consistent with earlier studies, which showed that the HSP90 co-chaperone SGT1 is required for auxin response in *Arabidopsis*^35^. To determine if HSP90 is required for auxin signalling apart from the temperature response, we examined the effect of GDA on a variety of auxin growth responses. First, we examined the effect of GDA on auxin-induced hypocotyl elongation. Treatment of light-grown seedlings with 2 μM IAA had a pronounced effect on hypocotyl elongation as previously reported (Fig. 2a)^39^. However, this effect was completely eliminated in the presence of 5 μM GDA. Similarly, the effect of IAA on lateral root formation and primary root growth was strongly suppressed by GDA (Fig. 2b–d). Finally, root gravitropism, an auxin-mediated process, was inhibited by GDA (Supplementary Fig. 4a). In addition, we found that radicicol also inhibited IAA-induced hypocotyl elongation and lateral root formation (Supplementary Fig. 4b,c). These results indicate that HSP90 is broadly required for auxin-dependent growth responses.

Auxin is known to act by regulating gene expression^40^. To determine if HSP90 is required for auxin-regulated transcription, we examined the effect of GDA on the auxin reporter *DR5:GUS*. As expected treatment of *DR5:GUS* seedlings with auxin resulted in a strong increase in GUS levels (Fig. 2e). Treatment with GDA substantially inhibited this response. We also examined the response of four auxin-regulated genes and obtained similar results. In the presence of GDA, the effect of IAA on gene expression was strongly inhibited. In the case of *IAA14,* the inhibition was almost complete (Supplementary Fig. 4d).

### HSP90 and high temperature stabilize the auxin co-receptors

**Figure 3:**
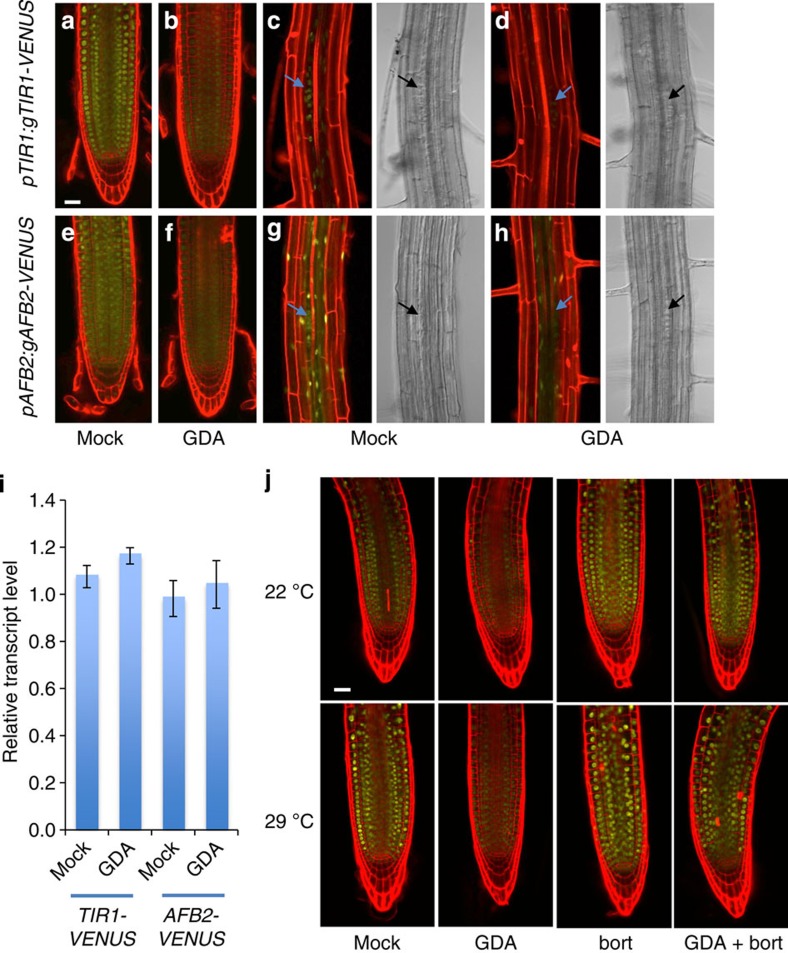
Inhibition of HSP90 destabilizes TIR1. (**a**–**h**) Five-day-old *pTIR1:gTIR1-VENUS* (**a**–**d**) and *pAFB2:gAFB2-VENUS* (**e**–**h**) seedlings were treated with dimethylsulphoxide (DMSO; **a**,**c**,**e**,**g**) or 10 μM GDA (G; **b**,**d**,**f**,**h**) for 24 h. The root tip (**a**,**b**,**e**,**f**) and root hair regions (**c**,**d**,**g**,**h**) of primary roots are shown. Arrows highlight lateral root primordia. (**i**) Relative levels of *TIR1-VENUS* and *AFB2-VENUS* transcripts (normalized to *GAPC2* mRNA level) were determined by qRT–PCR after treating Col-0 seedlings with GDA for various times. Error bars are s.d., *n*=3. (**j**) Five-day-old *pTIR1:gTIR1:VENUS* seedlings were treated with DMSO, 100 μM bortezomib (bort) or both 100 μM bortezomib and 10 μM GDA (G) and remained at 22 °C or shifted to 29 °C for 5 h before imaging. Scale bar, 50 μm.

Experiments in human cells have shown that HSP90 interacts with a large number of E3 ligases^34^ and in plants, the F-box protein ZEITLUPE (ZTL) is a client of HSP90 (ref. 32). Loss of HSP90 activity resulted in decreased stability of ZTL and a defect in circadian rhythm. As auxin is perceived by the TIR1/AFB auxin co-receptors, we wondered if these proteins might also be HSP90 clients. To test this possibility, we utilized *pTIR1:gTIR1-VENUS* and *pAFB2:gAFB2-VENUS* reporter lines. These lines were generated by amplifying the respective gene sequences, including introns and 4 kb of 5′ sequence, and placing *YFP* at the C-terminus of the gene. The constructs were introduced into *tir1-1* and *afb2-3* plants, respectively. Both *tir1-1* and *afb2-2* display modest resistance to IAA^36^ (Supplementary Fig. 5). Auxin response was restored by the transgenes (Supplementary Fig. 5) Confocal imaging of the root tips of *pTIR1:gTIR1-VENUS* and *pAFB2:gAFB2-VENUS* plants revealed that both proteins accumulate in most cell types in this region of the root and are primarily localized to the nucleus (Fig. 3a,e). When seedlings are treated with GDA for 24 h, there is a dramatic loss of TIR1-VENUS and AFB2-VENUS in all cell types even though this treatment does not result in a change in *TIR1* or *AFB2* transcript level (Fig. 3b,f,i). A similar effect was observed using a *pTIR1:cTIR1-GUS* line (Supplementary Fig 6c)^41^. Because HSP90 has many client proteins, it is possible that GUS or VENUS is destabilized by GDA. To exclude this possibility, we treated the *pTIR1:GUS* and *pUBQ10:CFP-VENUS* lines with GDA. The results shown in Supplementary Fig. 7a,b indicate that levels of the two reporter proteins are not affected by GDA.

As HSP90 is required for lateral root development, we also imaged lateral root primordia in the reporter lines. We have previously shown that TIR1 and AFB2 are required for lateral root formation^36^. As expected, the TIR1-VENUS and AFB2-VENUS proteins were observed in cells of stage II primordia (Fig. 3c,g)^42^. In contrast, very little VENUS signal is observed after GDA treatment (Fig. 3d,h). These results indicate that HSP90 is required for stability of TIR1 and AFB2 and further suggest that the effects of reduced HSP90 activity on auxin response are due to reduced levels of these auxin co-receptors.

We next asked if TIR1 degradation upon GDA treatment required the proteasome. *pTIR1:gTIR1-VENUS* seedlings were treated with GDA±the proteasome inhibitor bortezomib for 4 h before imaging. The results in Fig. 3j show that TIR1-VENUS levels were restored by bortezomib indicating that inhibition of HSP90 results in degradation of TIR1 by the proteasome.

**Figure 4:**
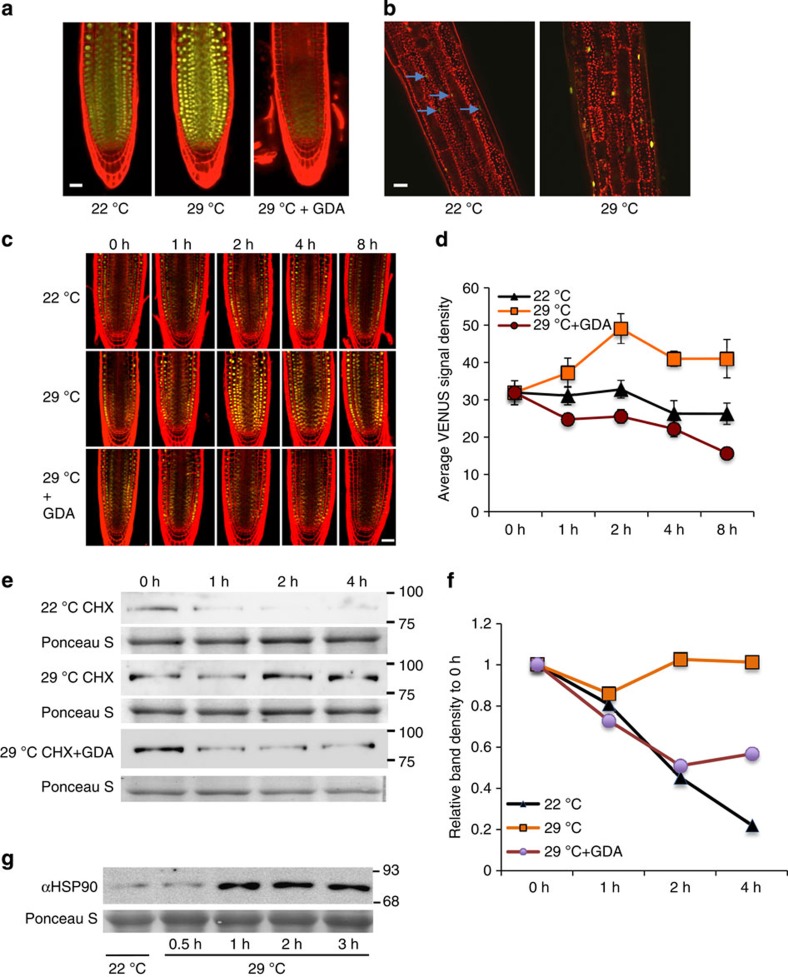
TIR1 levels rapidly increase in response to elevated temperature. (**a**,**b**) Five-day old *pTIR:gTIR1-VENUS* seedlings were shifted from 22 to 29 °C. The root tip (**a**) and the hypocotyl (**b**) were imaged after 24 h. In **a**, seedlings were also treated with 10 μM GDA (G). (**c**) Seedlings were treated as above and imaged at time intervals as shown. (**d**) Quantification of TIR1-VENUS levels from **c**. At least four seedlings were imaged for each time point. Error bars=s.d. (**e**) Five-day-old *pTIR1:gTIR1-VENUS* seedlings were transferred to 22 or 29 °C for 2 h and then treated with 200 μM CHX with our without 10 μM GDA at either 22 or 29 °C. Samples were collected at various time for protein blots. The experiment was repeated three times with similar results. (**f**) Quantitative protein blot band density is presented as relative to time zero of CHX treatment. (**g**) Five-day old Col-0 seedlings were shifted from 22 to 29 °C and HSP90 levels were determined by protein blot. Scale bars, 50 μm (**a**,**b**,**c**).

Our data suggest that HSP90 regulates the temperature growth response by enhancing auxin signalling rather than auxin biosynthesis (Fig. 1 and Supplementary Fig. 4). One possible mechanism is that high temperature promotes the accumulation of the TIR1/AFBs and that HSP90 is required for this process. To test this hypothesis, we first examined *TIR1, AFB2* and *AFB3* RNA levels in temperature-shifted seedlings by quantitative reverse transcription–PCR (qRT–PCR). The levels of these three transcripts were largely unchanged by the treatment (Supplementary Fig. 6b). We next examined TIR1-VENUS in the *pTIR1:gTIR1-VENUS* line after temperature shift. In this case, we observed a substantial increase in TIR1-VENUS levels in seedlings shifted to 29 °C (Fig. 4a). In the presence of GDA, this response was completely inhibited indicating that it requires HSP90. Similar results were obtained using the radiciol and ECGC (Supplementary Fig. 6a). As for seedlings at 22 °C, bortezomib restored TIR1-VENUS levels in the presence of GDA indicating that TIR1 is degraded by the proteasome at 29 °C as well (Fig. 3j). TIR1-VENUS levels were also strongly induced by 29 °C in the hypocotyl consistent with the growth response observed in this tissue at higher temperature (Fig. 4b). A similar result was observed in the *TIR1:cTIR1-GUS* plants, further demonstrating that the effect of high temperature and GDA is not on the green fluorescent protein (GFP) protein itself (Supplementary Fig. 6c). We next performed a time-course experiment to determine how quickly TIR1 levels changed. Remarkably, we found that TIR1 levels increase within 1 h and substantially increase within 2 h (Fig. 4c). Treatment with GDA strongly inhibited this response.

As elevated temperature does not affect transcription of the auxin co-receptor genes, it is possible that changes in stability are responsible for elevated TIR1 levels. To investigate this, we performed a cycloheximide experiment. The results in Fig. 4e,f show that TIR1-VENUS is substantially more stable at 29 °C than at 22 °C. The levels of TIR1 are essentially unchanged over 4 h at 29 °C, whereas they drop rapidly at 22 °C. In addition, GDA treatment reduced stability of TIR1-VENUS at 29 °C, indicating the increase in stability at elevated temperature is HSP90 dependent.

One possible explanation for the increased TIR1-VENUS stability at higher temperature is an increase in HSP90 levels. We extracted protein from seedlings shifted to 29 °C and determined HSP90 levels by protein blot. The results, shown in Fig. 4g, demonstrate that HSP90 levels are relatively low at 22 °C, but increase dramatically within 1 h of a shift to 29 °C coincident with increased TIR1-VENUS levels.

### TIR1 co-immunoprecipitates HSP90 and SGT1

**Figure 5:**
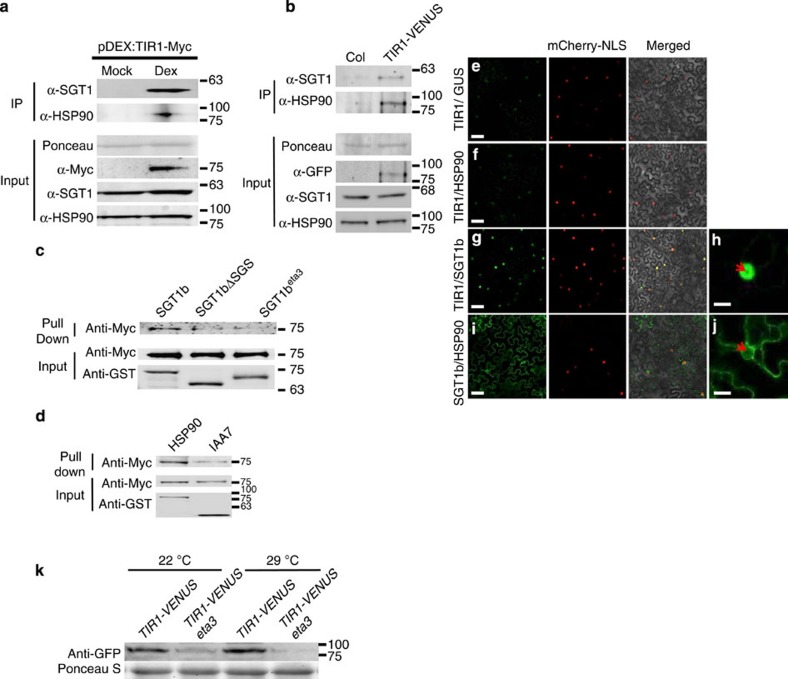
TIR1 is in a complex with HSP90 and SGT1. (**a**,**b**) Immunoprecipitation (IP) assays were performed using 7-day-old *pDEX:TIR1-Myc* (**a**) or *pTIR1:gTIR1-VENUS* (**b**) seedlings. TIR1, SGT1b and HSP90 were detected with the appropriate antibodies. (**c**) *In vitro* pull-down assay with GST-tagged SGT1b, SGT1bΔSGS and SGT1b(eta3). TIR1-Myc was synthesized in a TNT extract. (**d**) *In vitro* pull down with GST-tagged HSP90.2 and TIR1-Myc. GST-IAA7 was used as positive control. (**e**–**j**) YFP-fusion genes were introduced into *N. benthamiana* leaves by infiltration with *A. tumefaciens*. (**e**) nYFP-TIR1 and cYFP-GST (negative control). (**f**) nYFP-TIR1 and cYFP-HSP90. (**g**) nYFP-TIR1 and cYFP-SGT1b. (**i**) nYFP-SGT1b and cYFP-HSP90. (**h**,**j**) Enlarged images of single nuclei in (**g**) and (**i**) are shown, respectively. The arrows indicate the nucleolus. mCherry-NLS highlights nuclei. (**k**) Seven-day-old seedlings were transferred to 29 °C or kept at 22 °C for an additional 24 h before the tissue was harvested for protein blots. Scale bars, 50 μm (**e**,**f**,**g**,**i**) and 10 μm (**h**,**j**).

As HSP90 is required for TIR1 stability, it is possible that HSP90 interacts, directly or indirectly, with the auxin co-receptor. To test this possibility, we performed an immunoprecipitation experiment using *pDEX:TIR1-Myc* plants after dexamethasone treatment and tested for co-IP of HSP90. The *pDEX:TIR1-Myc* line was described previously^10^. The results in Fig. 5a show that HSP90 co-IPs with TIR1-Myc, indicating that HSP90 is present in a complex that includes TIR1. A similar experiment was done with the *pTIR1:gTIR1-VENUS* line and again HSP90 was recovered together with TIR1-VENUS (Fig. 5b).

The HSP90 co-chaperone SGT1 has been implicated in auxin signalling^25^. An *Arabidopsis* mutation in the *SGT1b* gene, called *eta3*, confers auxin resistance and reduced lateral root formation, defects that are similar to those observed in GDA-treated seedlings^25^. To determine if SGT1 also interacts with TIR1, we tested for co-IP with TIR1-Myc and TIR1-VENUS using an SGT1 antibody generated with a SGT1 peptide. The antibody recognizes both SGT1a and SGT1b in plant extracts (Supplementary Fig. 4). As seen in Fig. 5a,b, SGT1b is recovered with both TIR1-Myc and TIR1-VENUS.

To determine if HSP90 and/or SGT1b interact directly with TIR1, we performed a series of pull-down experiments. Glutathione *S*-transferase (GST)-tagged SGT1b and HSP90 synthesized in *Escherichia coli* were incubated with TIR1-myc synthesized in a TNT (Promega TNT Quick Coupled Transcription/Translation system) extract. The results in Fig. 5c,d show that TIR1-Myc is recovered in both the GST-SGT1b and GST-HSP90 pull-down indicating that both proteins interact directly with TIR1 in this assay. In previous studies, a region near the C-terminus of SGT1 called the SGT1-Specific (SGS) domain was shown to interact with the LRR domain of NLR proteins^31^. There is also evidence from other systems that this domain interacts with LRRs^31^,^43^. In addition, the *eta3* mutation results in a truncated protein lacking the C-terminal 36 amino acids of the SGS domain^25^. To determine if the SGS domain of SGT1b is required for interaction with TIR1, we repeated the pull-down experiment with mutant forms of SGT1b lacking the entire SGS domain or the last 36 amino acids as in *eta3*. We found that both mutant forms are deficient in TIR1-myc binding, indicating that the SGS domain is responsible for the interaction with TIR1.

To further explore the interaction between these proteins, we used the bimolecular fluorescence complementation (BiFC) system to test for interactions between TIR1, SGT1b and HSP90 in plant cells. cYFP and nYFP protein fusions were agro-infiltrated into tobacco leaf epidermal cells. Figure 5e is the negative control. The results clearly show that SGT1b interacted with HSP90 as expected (Fig. 5i,j) and that the interaction occurred in both the cytoplasm and the nucleus. In addition, we found that TIR1 interacted with SGT1b in the nucleus (Fig. 5g,h). However, we did not detect an interaction between TIR1 and HSP90 with this approach (Fig. 5f). These results are consistent with a recent report showing that TIR1 interacts with SGT1b, HSP90 and HSP70 in *Arabidopsis* protoplasts^44^.

As there are 5 *HSP90* genes encoding cytoplasmic forms of the protein, a genetic analysis of *HSP90* function is challenging. We attempted to knock down HSP90 levels using an artificial microRNA but the resulting lines were very unstable. As an alternative, we crossed the *pTIR1:gTIR1-VENUS* transgene into the *eta3* mutant and determined TIR1-VENUS levels by protein blot. The results in Fig. 5k show that TIR1-VENUS levels are reduced in *eta3* plants compared with the wild type at 22 °C. TIR1-VENUS levels increase at 29 °C in wild-type plants as anticipated. However, in the *eta3* background, this increase did not occur. These results provide genetic confirmation that the SGT1-HSP90 complex is required for TIR1 stability. In addition, they provide a clear molecular explanation for the auxin-resistant phenotype of the *eta3* mutant.

## Discussion

The TIR1/AFB auxin co-receptors promote degradation of the Aux/IAA transcriptional repressors in response to auxin. Although the regulation of Aux/IAA degradation has been extensively studied, we know little about the control of TIR1 stability or the role that changes in TIR1 level may have in auxin signalling. In a recent study, we demonstrated that TIR1 is an unstable protein^15^. Here we show that the molecular chaperone HSP90 acts to stabilize TIR1 and the related AFB2 protein. We demonstrate that TIR1 is in a complex with both HSP90 and the co-chaperone SGT1. Earlier genetic studies demonstrated that a mutation in *SGT1b* (*eta3*) resulted in both auxin and jasmonic acid (JA) resistance, but the basis for these defects was not clear^25^,^45^. We find that SGT1 binds directly to TIR1 *in vitro* through its SGS domain. This is consistent with reports that the SGS domain binds LRR domains in other client proteins including plant NLRs^43^,^46^,^47^. As the *eta3* mutation results in the loss of 36 amino acids at the C-terminus of SGT1b, including much of the SGS domain, our results provides a molecular explanation for the *eta3* auxin phenotype. Further, we find that the consequence of loss of SGT1b function in *eta3* is a reduction in TIR1 levels, consistent with a role for the SGT1-HSP90 co-chaperone–chaperone pair in TIR1 stability. A recent report demonstrated that HSP90 and SGT1b interact with the JA receptor COI1, an F-box protein closely related to TIR1 (ref. 44). A mutation in the *SGT1b* gene results in a reduction in COI1 levels and JA response. These workers also showed that SGT1b and HSP90 form a complex with TIR1 in protoplasts^44^. Interestingly, HSP90 has also been reported to function in brassinosteroid (BR) signalling^48^. In this case, HSP90 interacts with the BIN2 protein kinase and promotes nuclear localization. As BR is also involved in hypocotyl elongation and displays interactions with auxin, as well as other hormones, it is possible that changes in BR signalling may also contribute to the effects of GDA on auxin-induced hypocotyl growth. However, because the role of BR in the temperature response is less than that of auxin, and because we have shown that a number of other auxin response are affected by GDA, we think that a reduction in TIR1 levels is likely to be the primary cause of reduced hypocotyl elongation^35^. Further studies are required to clarify this issue. In any case, these results highlight the importance of the HSP90 system in hormone signalling in plants, as in animals.

The growth response of *Arabidopsis* hypocotyls to a mild increase in ambient temperature is well documented. According to the current model, the hypocotyl response is caused by the PIF4-regulated accumulation of auxin^7^,^8^. Our data indicate that this represents only part of the response and that HSP90-mediated increase in TIR1 levels is also required. We also show that elevation of ambient temperature from 22 to 29 °C has a broad effect on seedling growth with increased lateral root formation and primary root elongation. These changes are associated with a rapid increase in both TIR1 and HSP90 levels. HSP90 is required for maintaining TIR1 levels probably by protecting it from degradation through a proteasome-mediated pathway. The detailed mechanism of TIR1 stabilization at elevated temperature is unclear. It is likely that HSP90 helps to maintain TIR1 in its' native folded state. As the temperature rises, increased HSP90 further stabilizes TIR1 to promote growth. In this way, the HSP90-SGT1 chaperone system acts to integrate temperature and auxin signalling during seedling development.

The mechanism of HSP90 action is poorly understood but appears to be complex. The protein has a very flexible and dynamic structure and cooperates with many co-chaperones with diverse activities. Some co-chaperones act to regulate the ATPase activity of HSP90, but this is not the case with SGT1. Extensive studies of plant NLR proteins led to a model in which SGT1 binds to both the NLR protein, through the SGS domain, and the HSP90 dimer, via a second SGT1 domain called the CS (Chord and SGT1). In the case of NLRs, the RAR1 protein also binds to both HSP90 and SGT1, possibly acting to stabilize a functional complex. However, RAR1 is not required for auxin response suggesting that SGT1 is sufficient to promote the interaction between TIR1 and HSP90. Alternatively, an unknown protein may serve the function of RAR1. Finally, we note that HSP90 interacts directly with TIR1 *in vitro*. Although we were not able to demonstrate this interaction *in vivo,* it is possible that the inherent affinity of HSP90 for TIR1 overcomes the requirement for RAR1. In general, HSP90 is thought to act late in the protein folding pathway, including on completely folded proteins^49^,^50^. Because most of the 18 LRRs in TIR1 contribute to the auxin-binding pocket, HSP90, in concert with SGT1 may have an important role in maintaining the properly folded protein. Although our current model is that SGT1 acts in concert with HSP90 to regulate TIR1 folding and/or stability, it is also possible that SGT1 has a function that is independent of HSP90.

The responses of plants to a mild increase in ambient temperature and a heat shock are very different^5^. The former leads to increased plant growth, whereas the latter inhibits plant growth. Although HSFs and HSPs have been extensively studied in the context of a heat stress response, the function of these proteins in the response to mild temperature changes have not been reported. However, accumulating reports show that mutations or overexpression of HSFs or HSPs can strongly alter plant growth at normal temperatures, suggesting that these proteins may be important for plant growth in the absence of stress^51^,^52^,^53^,^54^,^55^,^56^. Our data suggest that in addition to their roles in heat stress response, the HSP and HSF pathways are also required for regulating plant growth in response to a mild increase in ambient temperature^2^. Therefore, these different temperature responses may share thermosensors and early signalling components. In natural conditions, the environmental temperature rises gradually rather than abruptly. It thus makes sense that plants have at least partially overlapping pathways to sense and respond to growth promoting temperatures and stressful heat shock. A mild increase in ambient temperature may promote plant growth and at the same time prime the plant for imminent heat stress. For example, a warm night or dawn may be indicative of upcoming heat at noon. Indeed, it has been reported that a gradual rise in temperature is more effective than an abrupt temperature increase in inducing acquired thermotolerance^2^,^57^.

## Methods

### Plant materials and growth conditions

*Arabidopsis thaliana* ecotype Col-0 was used in all experiments. The *pUBQ10:CFP-VENUS* line was provide by Julian Schroeder^58^. The *pTIR1:gTIR1-VENUS* and *pAFB2:gAFB2-VENUS* constructs were made by fusing in frame a genomic fragment consisting of a 4-kb promoter, the CDS of each gene and a *VENUS-*coding region in frame, followed by a 2-kb 3′ end region of each gene. Traditional cloning strategies including PCR, endonuclease digestion and ligation were adopted to introduce the fragments into a binary vector pGREENII 0229 (ref. 59). More details about the primers used for the cloning are shown in Supplementary Table 1. The resulting *pTIR1:gTIR1-VENUS* and *pAFB2:gAFB2-VENUS* were used to transform *tir1-1* and *afb2-3*, respectively^41^,^60^.

Sterilized seeds were sown on ½ MS media, and stratified at 4 °C for 3 days. All the hypocotyl and root assays were performed in short days (8 h light: 16 h dark) at a fluence rate of 60 μMol m^−2^ s^−1^. Except where described otherwise, seedlings were grown for 4 days before treatments.

### RNA extraction and qRT–PCR

Total RNA was extracted using RNA RNeasy Plant Mini Kits (Qiagen), and an on-column DNase digestion step was included during RNA extraction as recommended. cDNA was synthesized using SuperScript III (Life Technologies) and analysed by quantitative PCR on a CFX96 Real-Time System (Bio-Rad). RNA levels were normalized against transcripts of the *GAPC2* gene (AT1G13440). The oligos used for PCR are listed in Supplementary Table 1.

### GUS staining

For GUS staining seedlings were stained in 0.1 M sodium phosphate buffer (pH 7.0) containing 0.1% X-glc, 0.5 mM K_3_Fe(CN)6, 1 mM K_4_Fe(CN)_6_ and 0.05% Triton X-100 for 3 h followed by incubation in 70% (vol/vol) and then absolute ethanol for 1 h and overnight, respectively.

### Confocal microscopy

Seedlings were imaged on a laser scanning confocal microscope (Zeiss MLS 710). To visualize root anatomy, they were stained in 10 mg l^−1^ propidium iodide for 4–6 min, rinsed and mounted in water. Image J was used to quantify the intensity of yellow fluorescent protein (YFP) florescence signal in individual nuclei. Mean intensity was computed from all the nuclei in an image. Each image comprised 430 μm of the root tip measured from root cap, equivalent to one field of view with a × 20 objective lens. At least four images were used for each time point.

### Immunoprecipitation and *in vitro* pull-down experiments

For the immunoprecipitation assays, total proteins were extracted from 7-day-old *pGVG:TIR1-Myc* seedlings^10^ that were treated with or without 30 μM dexamethasone (DEX) for 24 h. Two milligrams of total proteins from each treatment were used for the immunoprecipitation. Four micrograms of anti-Myc monoclonal antibody (Covance, MMS-150R) was crosslinked by BS3 (Thermo Scientific Pierce, P121580) to Protein G Dynabeads (Life Technologies, 10004D) for 4 h at 4 °C. The complexes were washed five times and then applied to SDS–PAGE for protein blot detection. Anti-Myc (Roche, 11814150001 at 1:5,000 dilution), anti-HSP90 (Enzo Life Sciences AD1-SPA-835-F, 1:2,000) and anti-SGT1 (Customized anti sera. ProSci Incoporated, 1:5,000) were used in this study (Supplementary Fig. 8). The sequence of the antigen peptide used to raise the antibody was C-DWDKLEAEVKKQEKDEK. The TIR1-Myc co-IP experiment was repeated three times with similar results. For the co-IP experiments with TIR1-VENUS samples, the same experimental procedures were performed except 4 μg of anti-GFP antibody (Invitrogen, A11122) was used. The co-IP products and input samples were detected by anti-GFP (Invitrogen, A11122, at 1:2,500 dilution). anti-HSP90 (Enzo Life Sciences AD1-SPA-835-F, 1:2,000) and anti-SGT1 (Customized anti sera. ProSci Incoporated, 1:5,000).

To express recombinant proteins for *in vitro* pull-down assays, CDSs of *Arabidopsis* HSP90.2, IAA7, SGT1b, SGT1BΔSGS and SGT1b(eta3) were cloned into pENTR/D-Topo (Life Technologies, K240020SP) cloning vector and then into pDEST17 (Life Technologies) using GateWay cloning (Life Technologies, 11803-012). Primers used for the cloning are listed in Supplementary Table 1. GST-tagged proteins were purified from *E. coli* following a protocol described previously^15^. TIR1-Myc protein was synthesized *in vitro* using a TNT transcription and translation system. Equal amount of GST-tagged proteins were mixed with TIR1-Myc and incubated at 4 °C for 4 h followed by washing and protein blots to detect the recovered TIR1-Myc levels. Anti-Myc (Roche, 11814150001) at 1:5,000 dilution and anti-GST (Sigma-Aldrich, G7781-.2ML) at 1:5,000 dilution were used for the western blots. Original blot images are shown in Supplementary Fig. 9.

### Bimolecular florescence complementation

For the BiFC assay, sequences encoding TIR1, HSP90.2 and SGT1b were cloned into cloning vector *pENTR/D-Topo* (Life Technologies) and then destination vectors to make *nYFP-TIR1/SGT1b* and *cYFP-HSP90.2/SGT1b/GUS*, respectively. *pENTR-GUS* is from Life Technologies. Primers used for the cloning are listed in Supplementary Table 1. The *nYFP (pDEST–VYNE(R)*^*GW*^) and *cYFP (pDEST–VYCE(R)*^*GW*^) vectors are generous gifts from Dr Rainer Waadt and were described previously^61^. The resulting binary vectors carrying DNA sequences encoding the proteins fused to nYFP and cYFP were transferred into Agrobacteria strain GV3101 (pMP90). Equal amount of Agrobacteria carrying vectors for expressing nYFP- and cYFP-fusion proteins were mixed and *Nicotiana benthamiana* leaves were infiltrated with the Agrobacterium solution following a protocol as described previously^62^. Briefly, Agrobacteria carrying each construct were sedimented by centrifugation and resuspended in a solution of 10 mM MgCl2 and 150 μg ml^−1^ acetosyringone to give a final OD600 of 0.8. The cells were then incubated at room temperature for 3 h before being infiltrated into the abaxial surface of ∼4-week-old *N. benthamiana* leaves.

## Additional information

**How to cite this article:** Wang, R. *et al.* HSP90 regulates temperature-dependent seedling growth in *Arabidopsis* by stabilizing the auxin co-receptor F-box protein TIR1. *Nat. Commun.* 7:10269 doi: 10.1038/ncomms10269 (2016).

## Supplementary Material

Supplementary Information Supplementary Figures 1-9 and Supplementary Table 1

## References

[b1] Kotak S. *et al.* Complexity of the heat stress response in plants. Curr. Opin. Plant Biol. 10, 310–316 (2007). 17482504 10.1016/j.pbi.2007.04.011

[b2] Mittler R., Finka A. & Goloubinoff P. How do plants feel the heat? Trends Biochem. Sci. 37, 118–125 (2012). 22236506 10.1016/j.tibs.2011.11.007

[b3] Fragkostefanakis S., RÖTh S., Schleiff E. & Scharf K.-D. Prospects of engineering thermotolerance in crops through modulation of heat stress transcription factor and heat shock protein networks. Plant Cell Environ. 38, 1881–1895 (2014). 24995670 10.1111/pce.12396

[b4] Xu J. *et al.* Overexpression of GmHsp90s, a Heat Shock Protein 90 (Hsp90) Gene Family Cloning from Soybean, Decrease Damage of Abiotic Stresses in *Arabidopsis* thaliana. PLoS ONE 8, e69810 (2013). 23936107 10.1371/journal.pone.0069810 PMC3723656

[b5] Wigge P. A. Ambient temperature signalling in plants. Curr. Opin. Plant Biol. 16, 661–666 (2013). 24021869 10.1016/j.pbi.2013.08.004

[b6] Peng S. *et al.* Rice yields decline with higher night temperature from global warming. Proc. Natl Acad. Sci. USA 101, 9971–9975 (2004). 15226500 10.1073/pnas.0403720101 PMC454199

[b7] Franklin K. A. *et al.* Phytochrome-interacting factor 4 (PIF4) regulates auxin biosynthesis at high temperature. Proc. Natl Acad. Sci. USA 108, 20231–20235 (2011). 22123947 10.1073/pnas.1110682108 PMC3250122

[b8] Sun J., Qi L., Li Y., Chu J. & Li C. PIF4-mediated activation of YUCCA8 expression integrates temperature into the auxin pathway in regulating *Arabidopsis* hypocotyl growth. PLoS Genet. 8, e1002594 (2012). 22479194 10.1371/journal.pgen.1002594 PMC3315464

[b9] Kumar S. V. & Wigge P. A. H2A.Z-containing nucleosomes mediate the thermosensory response in *Arabidopsis*. Cell 140, 136–147 (2010). 20079334 10.1016/j.cell.2009.11.006

[b10] Gray W. M., Kepinski S., Rouse D., Leyser O. & Estelle M. Auxin regulates SCF(TIR1)-dependent degradation of AUX/IAA proteins. Nature 414, 271–276 (2001). 11713520 10.1038/35104500

[b11] Kepinski S. & Leyser O. The *Arabidopsis* F-box protein TIR1 is an auxin receptor. Nature 435, 446–451 (2005). 15917798 10.1038/nature03542

[b12] Dharmasiri N., Dharmasiri S. & Estelle M. The F-box protein TIR1 is an auxin receptor. Nature 435, 441–445 (2005). 15917797 10.1038/nature03543

[b13] Tan X. *et al.* Mechanism of auxin perception by the TIR1 ubiquitin ligase. Nature 446, 640–645 (2007). 17410169 10.1038/nature05731

[b14] Calderon Villalobos L. I. *et al.* A combinatorial TIR1/AFB-Aux/IAA co-receptor system for differential sensing of auxin. Nat. Chem. Biol. 8, 477–485 (2012). 22466420 10.1038/nchembio.926 PMC3331960

[b15] Yu H. *et al.* Untethering the TIR1 auxin receptor from the SCF complex increases its stability and inhibits auxin response. Nat. Plants 1, pii 14030 (2015). 10.1038/nplants.2014.30 PMC4520256 26236497

[b16] Duda D. M. *et al.* Structural regulation of cullin-RING ubiquitin ligase complexes. Curr. Opin. Struct. Biol. 21, 257–264 (2011). 21288713 10.1016/j.sbi.2011.01.003 PMC3151539

[b17] Enchev R. I. *et al.* Structural basis for a reciprocal regulation between SCF and CSN. Cell Rep. 2, 616–627 (2012). 22959436 10.1016/j.celrep.2012.08.019 PMC3703508

[b18] Pierce N. W. *et al.* Cand1 promotes assembly of new SCF complexes through dynamic exchange of F box proteins. Cell 153, 206–215 (2013). 23453757 10.1016/j.cell.2013.02.024 PMC3656483

[b19] Chuang H. W., Zhang W. & Gray W. M. *Arabidopsis* ETA2, an apparent ortholog of the human cullin-interacting protein CAND1, is required for auxin responses mediated by the SCF(TIR1) ubiquitin ligase. Plant Cell 16, 1883–1897 (2004). 15208392 10.1105/tpc.021923 PMC514168

[b20] del Pozo J. C. & Estelle M. The *Arabidopsis* cullin AtCUL1 is modified by the ubiquitin-related protein RUB1. Proc. Natl Acad. Sci. USA 96, 15342–15347 (1999). 10611386 10.1073/pnas.96.26.15342 PMC24821

[b21] Schwechheimer C. *et al.* Interactions of the COP9 signalosome with the E3 ubiquitin ligase SCFTIRI in mediating auxin response. Science 292, 1379–1382 (2001). 11337587 10.1126/science.1059776

[b22] Hua Z., Zou C., Shiu S. H. & Vierstra R. D. Phylogenetic comparison of F-Box (FBX) gene superfamily within the plant kingdom reveals divergent evolutionary histories indicative of genomic drift. PLoS ONE 6, e16219 (2011). 21297981 10.1371/journal.pone.0016219 PMC3030570

[b23] Gray W. M. *et al.* Identification of an SCF ubiquitin-ligase complex required for auxin response in *Arabidopsis* thaliana. Genes Dev 13, 1678–1691 (1999). 10398681 10.1101/gad.13.13.1678 PMC316846

[b24] Hellmann H. *et al.* *Arabidopsis* AXR6 encodes CUL1 implicating SCF E3 ligases in auxin regulation of embryogenesis. EMBO J. 22, 3314–3325 (2003). 12839993 10.1093/emboj/cdg335 PMC165659

[b25] Gray W. M., Muskett P. R., Chuang H. W. & Parker J. E. *Arabidopsis* SGT1b is required for SCF(TIR1)-mediated auxin response. Plant Cell 15, 1310–1319 (2003). 12782725 10.1105/tpc.010884 PMC156368

[b26] Catlett M. G. & Kaplan K. B. Sgt1p is a unique co-chaperone that acts as a client adaptor to link Hsp90 to Skp1p. J. Biol. Chem. 281, 33739–33748 (2006). 16945921 10.1074/jbc.M603847200

[b27] Zhang M. *et al.* Structural and functional coupling of Hsp90- and Sgt1-centred multi-protein complexes. EMBO J. 27, 2789–2798 (2008). 18818696 10.1038/emboj.2008.190 PMC2556094

[b28] Shirasu K. The HSP90-SGT1 chaperone complex for NLR immune sensors. Annu. Rev. Plant Biol. 60, 139–164 (2009). 19014346 10.1146/annurev.arplant.59.032607.092906

[b29] Takahashi A., Casais C., Ichimura K. & Shirasu K. HSP90 interacts with RAR1 and SGT1 and is essential for RPS2-mediated disease resistance in *Arabidopsis*. Proc. Natl Acad. Sci. USA 100, 11777–11782 (2003). 14504384 10.1073/pnas.2033934100 PMC208834

[b30] Pearl L. H. & Prodromou C. Structure and mechanism of the Hsp90 molecular chaperone machinery. Annu. Rev. Biochem. 75, 271–294 (2006). 16756493 10.1146/annurev.biochem.75.103004.142738

[b31] Kadota Y., Shirasu K. & Guerois R. NLR sensors meet at the SGT1-HSP90 crossroad. Trends Biochem. Sci. 35, 199–207 (2010). 20096590 10.1016/j.tibs.2009.12.005

[b32] Kim T. S. *et al.* HSP90 functions in the circadian clock through stabilization of the client F-box protein ZEITLUPE. Proc. Natl Acad. Sci. USA 108, 16843–16848 (2011). 21949396 10.1073/pnas.1110406108 PMC3189077

[b33] Taipale M., Jarosz D. F. & Lindquist S. HSP90 at the hub of protein homeostasis: emerging mechanistic insights. Nat. Rev. Mol. Cell Biol. 11, 515–528 (2010). 20531426 10.1038/nrm2918

[b34] Taipale M. *et al.* Quantitative analysis of HSP90-client interactions reveals principles of substrate recognition. Cell 150, 987–1001 (2012). 22939624 10.1016/j.cell.2012.06.047 PMC3894786

[b35] Gray W. M., Ostin A., Sandberg G., Romano C. P. & Estelle M. High temperature promotes auxin-mediated hypocotyl elongation in *Arabidopsis*. Proc. Natl Acad. Sci. USA 95, 7197–7202 (1998). 9618562 10.1073/pnas.95.12.7197 PMC22781

[b36] Dharmasiri N. *et al.* Plant development is regulated by a family of auxin receptor F box proteins. Dev. Cell 9, 109–119 (2005). 15992545 10.1016/j.devcel.2005.05.014

[b37] Yin Z., Henry E. C. & Gasiewicz T. A. (-)-Epigallocatechin-3-gallate is a novel Hsp90 inhibitor. Biochemistry 48, 336–345 (2009). 19113837 10.1021/bi801637q PMC2701625

[b38] Sun J., Qi L., Li Y., Chu J. & Li C. PIF4–Mediated Activation of *YUCCA8* Expression Integrates Temperature into the Auxin Pathway in Regulating *Arabidopsis* Hypocotyl Growth. PLoS Genet. 8, e1002594 (2012). 22479194 10.1371/journal.pgen.1002594 PMC3315464

[b39] Chapman E. J. *et al.* Hypocotyl transcriptome reveals auxin regulation of growth-promoting genes through GA-dependent and -independent pathways. PLoS ONE 7, e36210 (2012). 22590525 10.1371/journal.pone.0036210 PMC3348943

[b40] Chapman E. J. & Estelle M. Mechanism of auxin-regulated gene expression in plants. Annu. Rev. Genet. 43, 265–285 (2009). 19686081 10.1146/annurev-genet-102108-134148

[b41] Parry G. *et al.* Complex regulation of the TIR1/AFB family of auxin receptors. Proc. Natl Acad. Sci. USA 106, 22540–22545 (2009). 20018756 10.1073/pnas.0911967106 PMC2799741

[b42] Peret B. *et al.* *Arabidopsis* lateral root development: an emerging story. Trends Plant Sci. 14, 399–408 (2009). 19559642 10.1016/j.tplants.2009.05.002

[b43] Leister R. T. *et al.* Molecular genetic evidence for the role of SGT1 in the intramolecular complementation of Bs2 protein activity in Nicotiana benthamiana. Plant Cell 17, 1268–1278 (2005). 15749757 10.1105/tpc.104.029637 PMC1088001

[b44] Zhang X.-C., Millet Y. A., Cheng Z., Bush J. & Ausubel F. M. Jasmonate signalling in *Arabidopsis* involves SGT1b–HSP70–HSP90 chaperone complexes. Nat. Plants 1, pii 15049 (2015). 10.1038/nplants.2015.49 PMC4819967 27054042

[b45] He Y., Chung E. H., Hubert D. A., Tornero P. & Dangl J. L. Specific missense alleles of the *Arabidopsis* jasmonic acid co-receptor COI1 regulate innate immune receptor accumulation and function. PLoS Genet. 8, e1003018 (2012). 23093946 10.1371/journal.pgen.1003018 PMC3475666

[b46] Bieri S. *et al.* RAR1 positively controls steady state levels of barley MLA resistance proteins and enables sufficient MLA6 accumulation for effective resistance. Plant Cell 16, 3480–3495 (2004). 15548741 10.1105/tpc.104.026682 PMC535887

[b47] Dubacq C., Guerois R., Courbeyrette R., Kitagawa K. & Mann C. Sgt1p contributes to cyclic AMP pathway activity and physically interacts with the adenylyl cyclase Cyr1p/Cdc35p in budding yeast. Eukaryot. Cell 1, 568–582 (2002). 12456005 10.1128/EC.1.4.568-582.2002 PMC118006

[b48] Samakovli D., Margaritopoulou T., Prassinos C., Milioni D. & Hatzopoulos P. Brassinosteroid nuclear signaling recruits HSP90 activity. New Phytol. 203, 743–757 (2014). 24807419 10.1111/nph.12843

[b49] Karagöz G. E. & Rüdiger S. G. D. Hsp90 interaction with clients. Trends Biochem. Sci. 40, 117–125 (2015). 25579468 10.1016/j.tibs.2014.12.002

[b50] Saibil H. Chaperone machines for protein folding, unfolding and disaggregation. Nat. Rev. Mol. Cell Biol. 14, 630–642 (2013). 24026055 10.1038/nrm3658 PMC4340576

[b51] Ikeda M., Mitsuda N. & Ohme-Takagi M. *Arabidopsis* HsfB1 and HsfB2b act as repressors of the expression of heat-inducible Hsfs but positively regulate the acquired thermotolerance. Plant Physiol. 157, 1243–1254 (2011). 21908690 10.1104/pp.111.179036 PMC3252156

[b52] Kolmos E., Chow B. Y., Pruneda-Paz J. L. & Kay S. A. HsfB2b-mediated repression of PRR7 directs abiotic stress responses of the circadian clock. Proc. Natl Acad. Sci. 111, 16172–16177 (2014). 25352668 10.1073/pnas.1418483111 PMC4234549

[b53] Ogawa D., Yamaguchi K. & Nishiuchi T. High-level overexpression of the *Arabidopsis* HsfA2 gene confers not only increased themotolerance but also salt/osmotic stress tolerance and enhanced callus growth. J. Exp. Botany 58, 3373–3383 (2007). 17890230 10.1093/jxb/erm184

[b54] Noël L. D. *et al.* Interaction between SGT1 and cytosolic/nuclear HSC70 chaperones regulates *Arabidopsis* immune responses. Plant Cell 19, 4061–4076 (2007). 18065690 10.1105/tpc.107.051896 PMC2217652

[b55] Jungkunz I. *et al.* AtHsp70-15-deficient *Arabidopsis* plants are characterized by reduced growth, a constitutive cytosolic protein response and enhanced resistance to TuMV. Plant J. 66, 983–995 (2011). 21418353 10.1111/j.1365-313X.2011.04558.x

[b56] Liu H.-c. & Charng Y.-y. Common and distinct functions of *Arabidopsis* Class A1 and A2 heat shock factors in diverse abiotic stress responses and development. Plant Physiol. 163, 276–290 (2013). 23832625 10.1104/pp.113.221168 PMC3762648

[b57] Larkindale J. & Vierling E. Core genome responses involved in acclimation to high temperature. Plant Physiol. 146, 748–761 (2008). 18055584 10.1104/pp.107.112060 PMC2245833

[b58] Waadt R., Schlucking K., Schroeder J. I. & Kudla J. Protein fragment bimolecular fluorescence complementation analyses for the *in vivo* study of protein-protein interactions and cellular protein complex localizations. Methods Mol. Biol. 1062, 629–658 (2014). 24057390 10.1007/978-1-62703-580-4_33 PMC4073779

[b59] Hellens R. P., Edwards E. A., Leyland N. R., Bean S. & Mullineaux P. M. pGreen: a versatile and flexible binary Ti vector for Agrobacterium-mediated plant transformation. Plant Mol. Biol. 42, 819–832 (2000). 10890530 10.1023/a:1006496308160

[b60] Ruegger M. *et al.* The TIR1 protein of *Arabidopsis* functions in auxin response and is related to human SKP2 and yeast grr1p. Genes Dev. 12, 198–207 (1998). 9436980 10.1101/gad.12.2.198 PMC316440

[b61] Gehl C., Waadt R., Kudla J., Mendel R. R. & Hansch R. New GATEWAY vectors for high throughput analyses of protein-protein interactions by bimolecular fluorescence complementation. Mol. plant 2, 1051–1058 (2009). 19825679 10.1093/mp/ssp040

[b62] Voinnet O., Rivas S., Mestre P. & Baulcombe D. An enhanced transient expression system in plants based on suppression of gene silencing by the p19 protein of tomato bushy stunt virus. Plant J. Cell Mol. Biol. 33, 949–956 (2003). 10.1046/j.1365-313x.2003.01676.x 12609035

