# Corrigendum: HSP90 regulates temperature-dependent seedling growth in *Arabidopsis* by stabilizing the auxin co-receptor F-box protein TIR1

**DOI:** 10.1038/ncomms11677

**Published:** 2016-05-11

**Authors:** Renhou Wang, Yi Zhang, Martin Kieffer, Hong Yu, Stefan Kepinski, Mark Estelle

Nature Communications 7: Article number: 10269 10.1038/ncomms10269 (2016); Published: 01052015; Updated: 05112016

This Article contains errors in the labelling of the *x* axis in Supplementary Figure 3 (lower panel). The *x* axis in this panel should have been labelled ‘22 °C Mock; 22 °C GDA; 29 °C Mock; 29 °C GDA'. The correct version of this figure appears below.


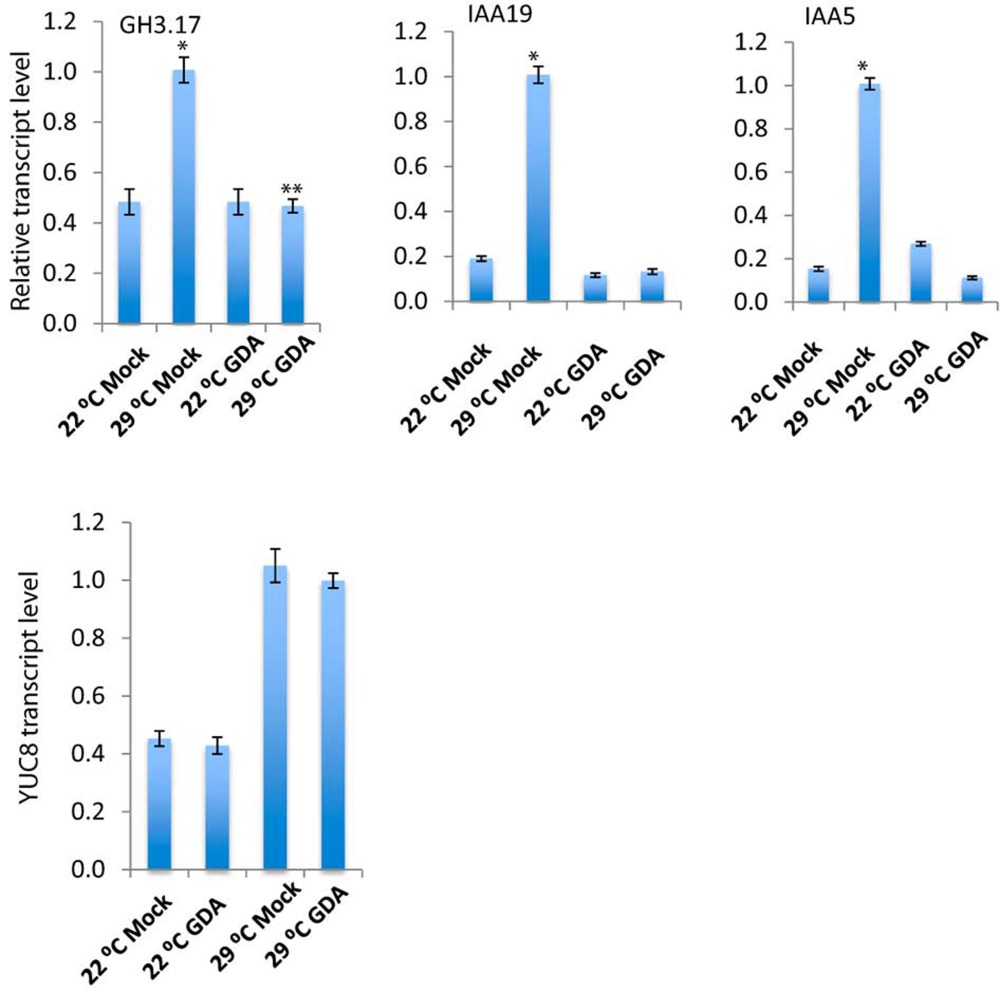


**Supplementary Figure 3**

