# Supplementary material for: HSP90 regulates temperature-dependent seedling growth in Arabidopsis by stabilizing the auxin co-receptor F-box protein TIR1
Source: Nat Commun. 2016 Jan 5;7:10269. doi: 10.1038/ncomms10269 (PMC4728404; doi:10.1038/ncomms10269)
Supplement: Supplementary Information — Supplementary Figures 1-9 and Supplementary Table 1 [file ncomms10269-s1.pdf]

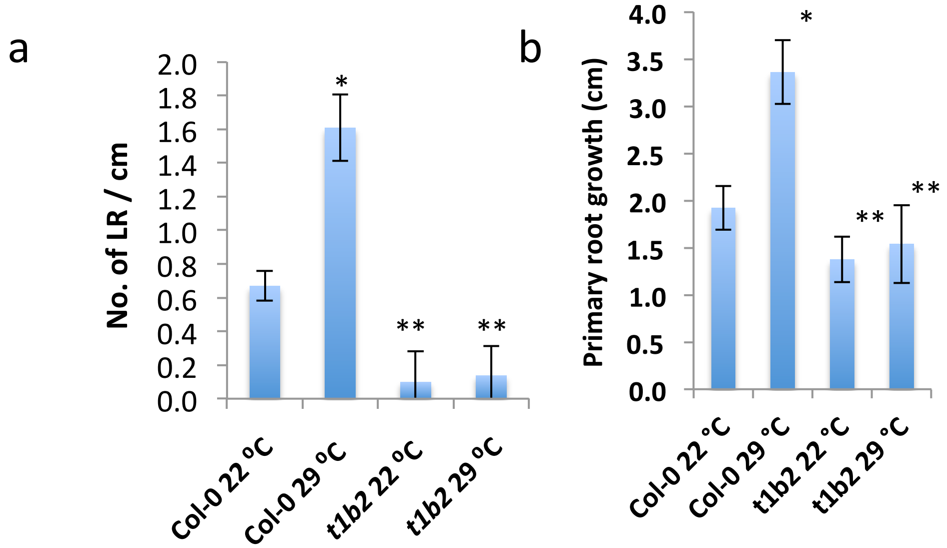

Supplementary Fig 1. Role of TIR1 and AFB2 in seedling growth at elevated temperature. (a) and (b) 5 day-old seedlings were maintained at 22 ^{0}C or shifted to 29 ^{0}C. The number of lateral roots (a) and length of primary roots (b) were measured after 4 more days. *t1b1* is *tir1-1 afb2-3*. Error bars are SD., n=12-15 seedlings, * significant difference with 22^{0}C, **significant difference with Col-0 (t-test P<0.01).

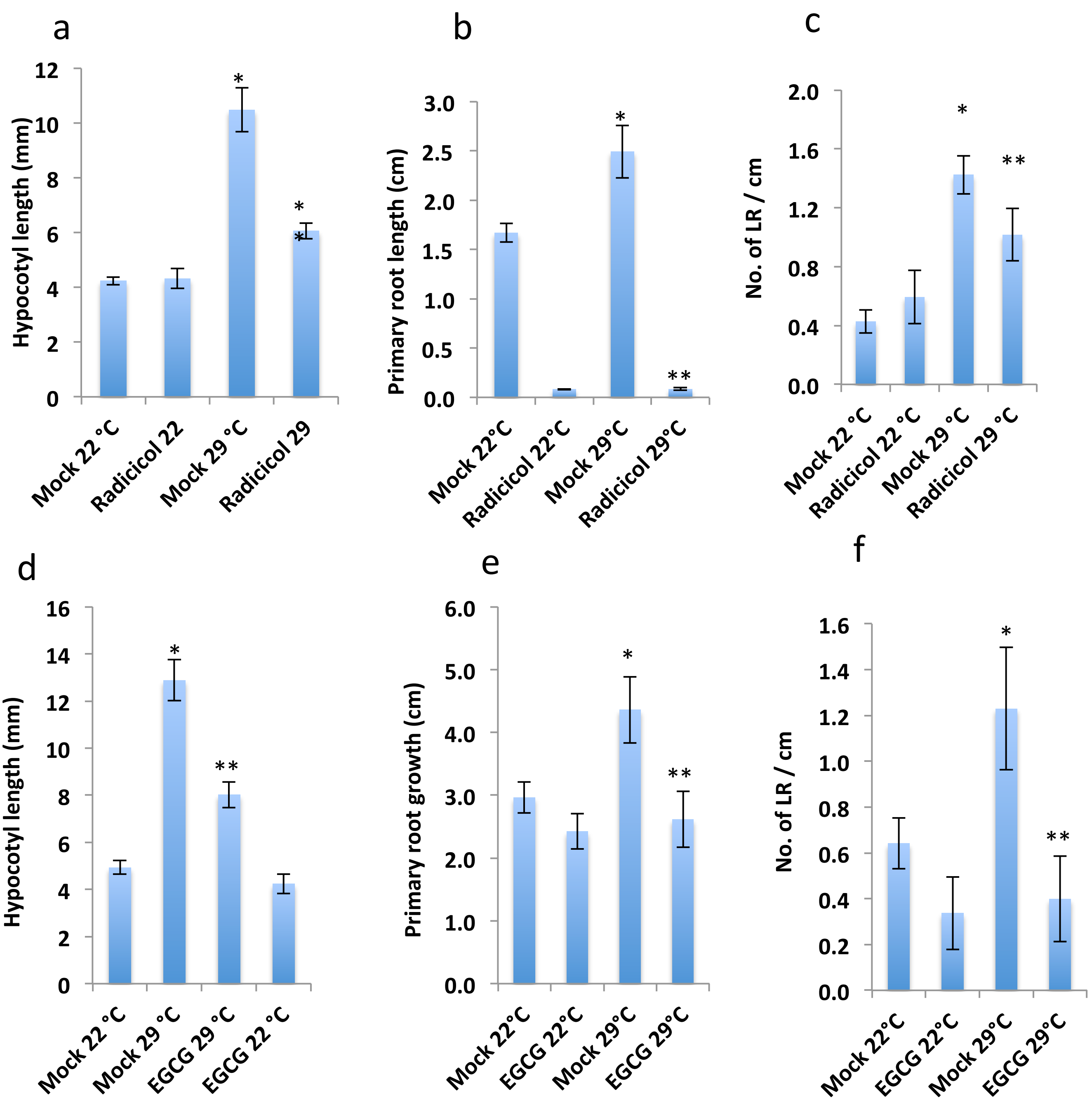

Supplementary Fig 2 Effects of the HSP90 inhibitor Radiciol and EGCG on seedling growth at elevated temperature. (a) and (d) 5 day-old seedlings were maintained at 22 ^{0}C or shifted to 29 ^{0}C in the presence or absence of 10 µM Radicicol or 100 µM EGCG and grown for another 4 days. (b) (c) (e) (f) 7 day-old seedlings were maintained at 22 ^{0}C or shifted to 29 ^{0}C in the presence or absence of 2 µM Radicicol or 25 µM EGCG and grown for another 6 days. Error bars are SD. N=10-14. * significant difference with 22°C, **significant difference with Col-0 (t-test *P*<0.01

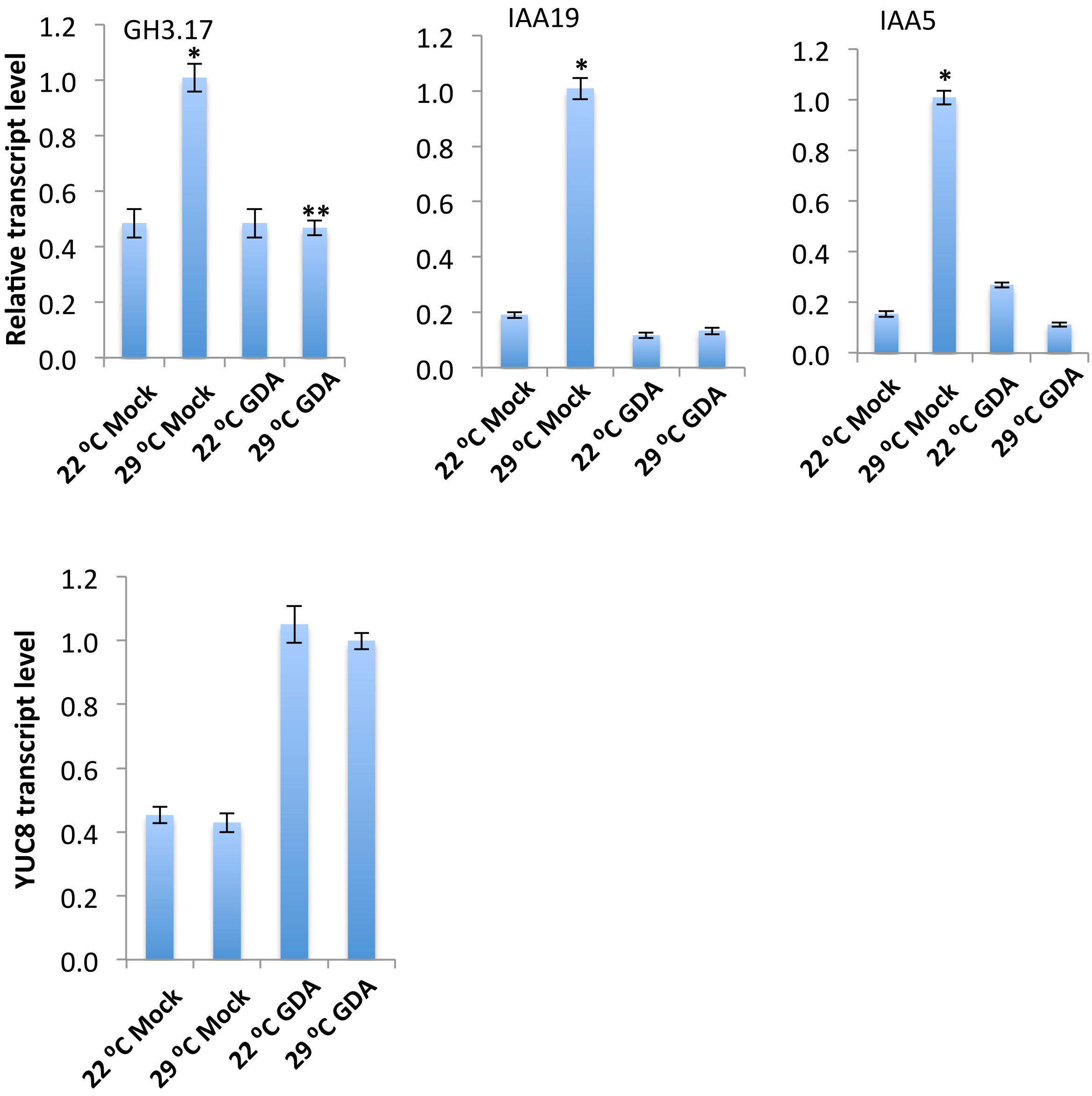

Supplementary Fig 3. Effect of GDA (G) treatment on temperature-regulated gene expression. 5-day old Col-0 seedlings were shifted to 29 ^{0}C. RNA levels were determined by qRT-PCR after 24 hours. Error bars are SD. N=3. * significant difference with 22 ^{0}C, ** significant difference with mock

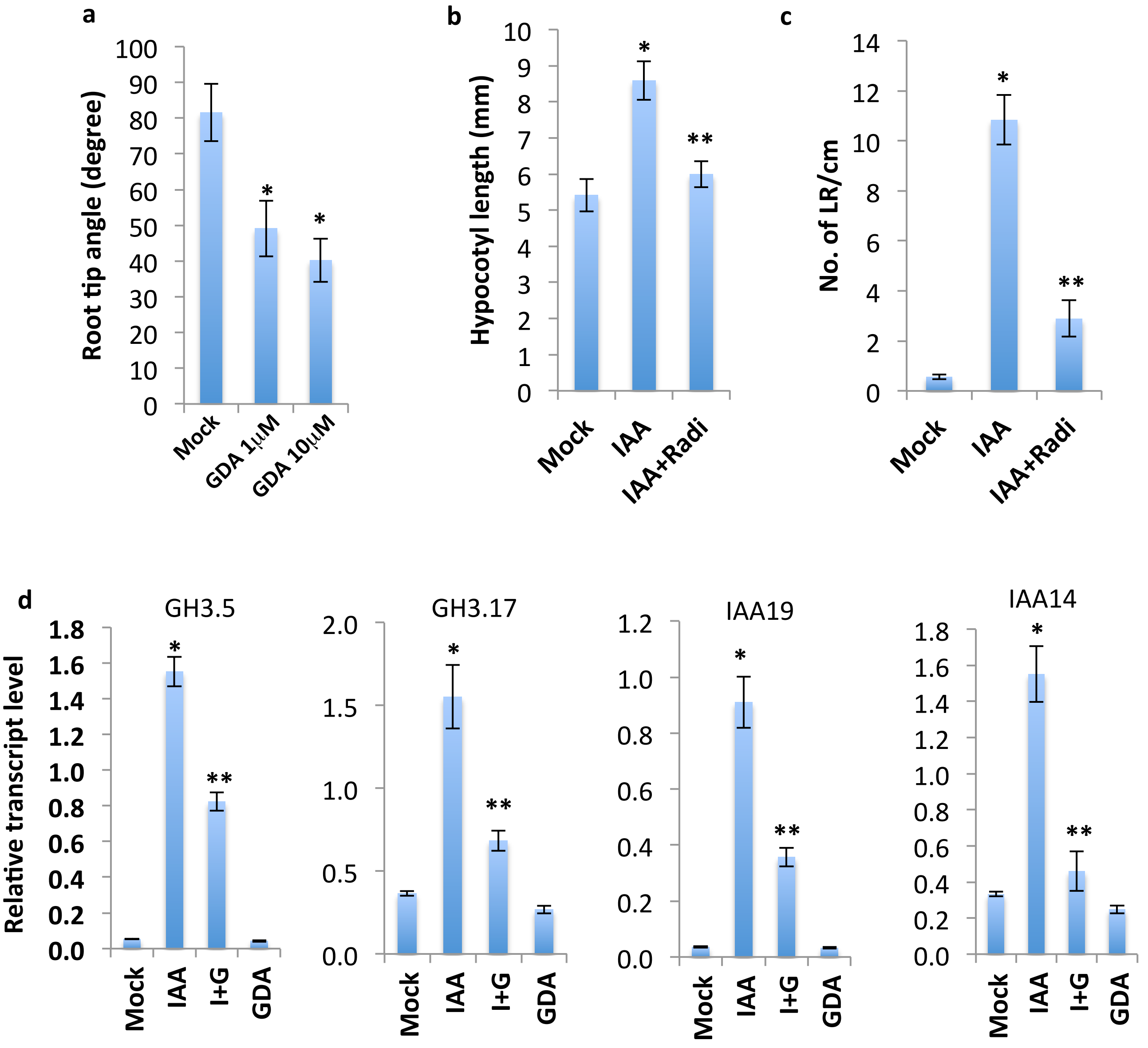

Supplementary Fig 4. Inhibition of HSP90 activity suppresses response of seedlings to auxin. (a) Root gravitropism was reduced after being treated with GDA. Seedlings were grown on ½ MS for 5 days, and then transferred to ½ MS plus GDA or DMSO (Mock). After growth of the seedlings for 2 hours in the vertical orientation, the plates were turned 90° and the root tip angle was measured after 24 hours. (b, c) Col-0 seedlings were treated with IAA (2 μM), Radicicol (Radi, 10 μM) or both for 5 days. Error bars are SD. * significantly different from mock, ** significantly different from IAA (t-test $P<0.01$) (d) Five day old Col-0 seedlings were treated with IAA (2 μM), GDA (10 μM) or both for 24 hours. Transcript levels (*GAPC2* mRNA level were used for normalization) were determined by qRT-PCR. Error bars are SD., n=3. * significant difference from mock, ** significant difference from IAA (t-test $P<0.01$).

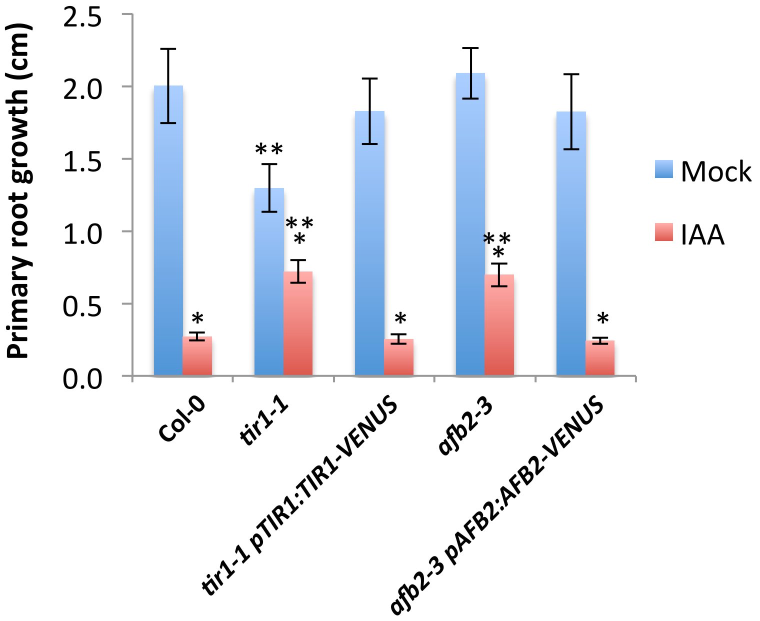

Supplementary Fig 5. The *pTIR1:TIR1-VENUS* and *pAFB2:AFB2-VENUS* complement the *tir1-1* and *afb2-3* mutants, respectively. 5-day old seedlings were treated with 0.02 µM IAA or DMSO (Mock) for another 5 days before the length of the newly grown primary roots were measured. Error bars are SD., n=12-14 * significant difference with mock, ** significant difference with Col-0.

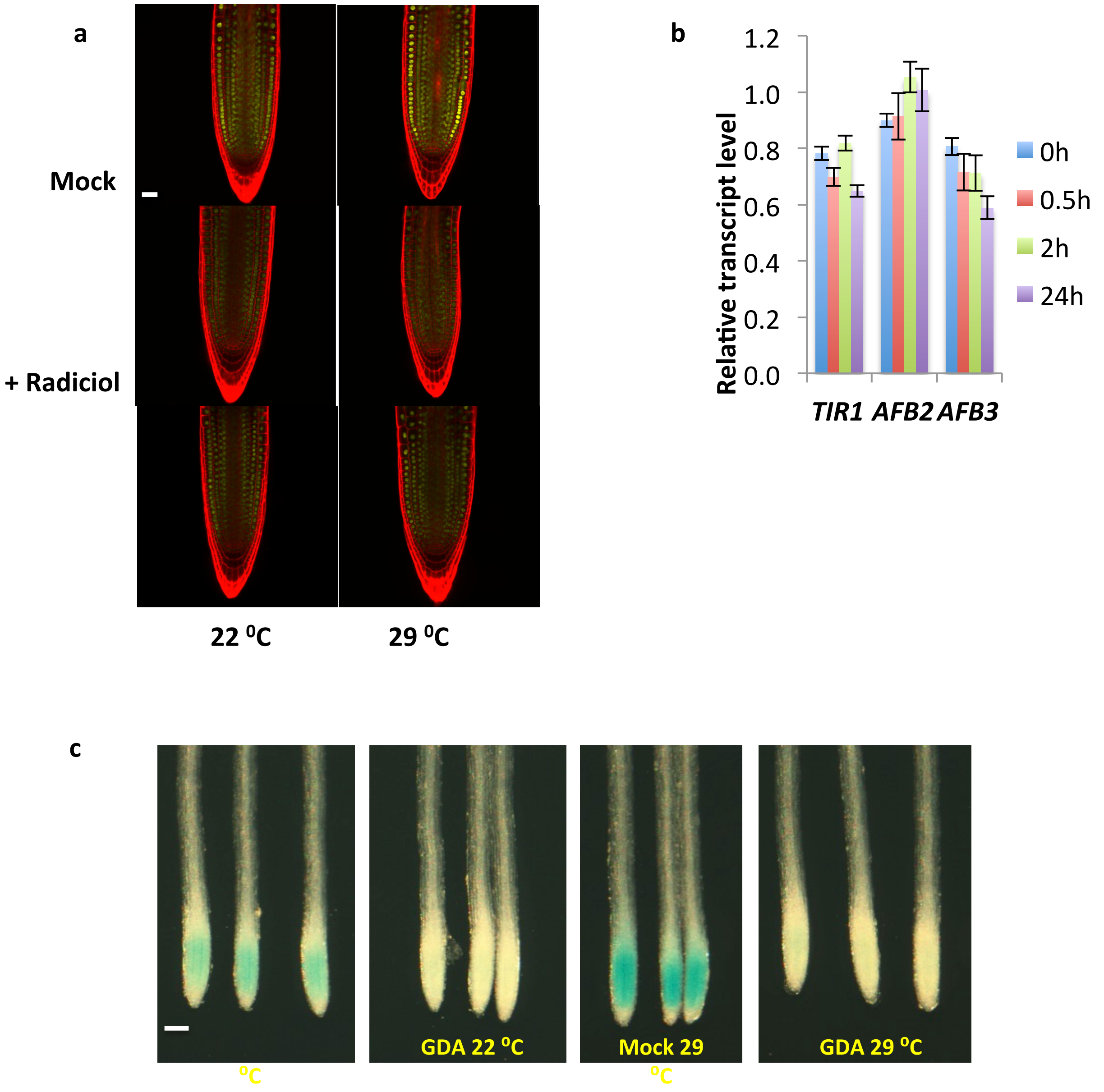

Supplementary Fig 6. Effect of treatments on auxin receptor levels. (a) 5 day-old *pTIR1:TIR1-GUS* seedlings were either maintained at 22 °C or shifted to 29 °C in the absence or presence of 100 mM GDA(G) and stained for GUS after 24 hours (b) 5 day-old Col-0 seedlings were transferred to 29 °C and *TIR1/AFB* transcript levels were determined by qRT-PCR at various times. Error bars are SD. Scale bars represent 50 (a) and 200 (c) µm, respectively.

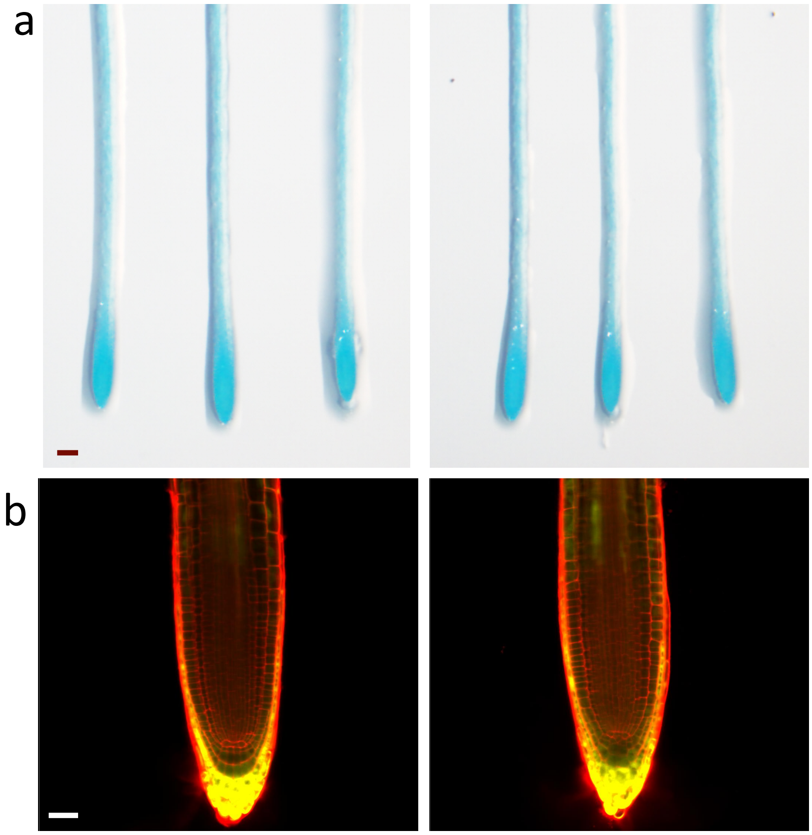

Supplementary Figure 7. (a) 5 day-old *pTIR1:TIR1-GUS* seedlings were treated with DMSO or 10μM GDA for 9 hours before staining for GUS. (b) 4 day-old *pUBQ10:CFP-VENUS* seedlings were treated with DMSO or 10 μM GDA for 11 hours before imaging by confocal microscopy. Scale bar (a) 200 μm; (b) 50 μm

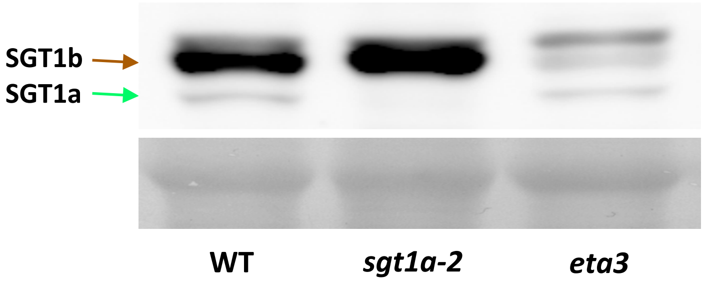

Supplementary Fig 8. **Characterization of SGT1 antibody.** Custom anti-SGT1 antibody recognizes both SGT1a and SGT1b in protein extracted from Col-0, *sgt1a-2* and *eta3* seedlings.

Supplementary Fig 9. Original Blots

Figure 4e

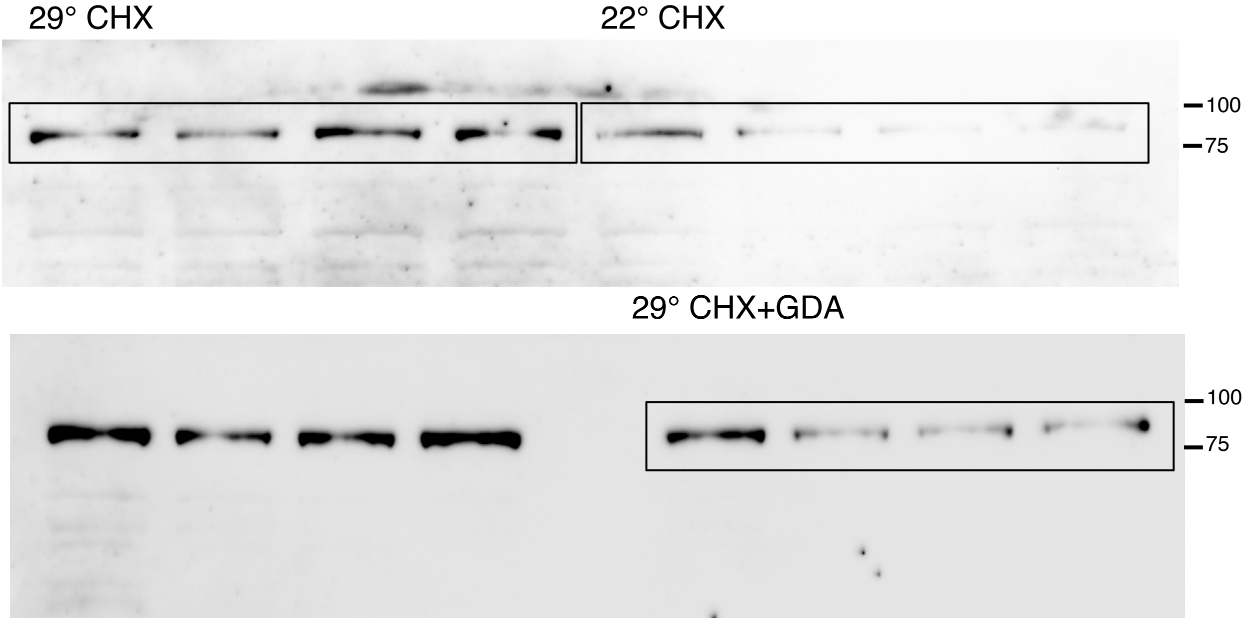

Fig. 4g

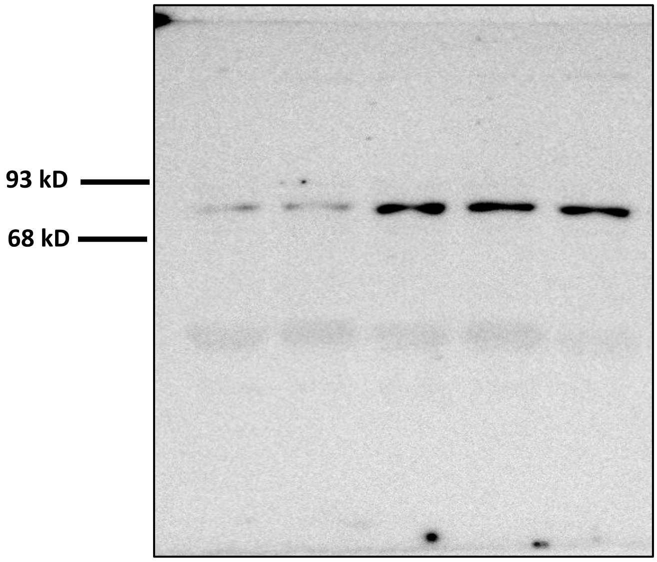

Figure 5a

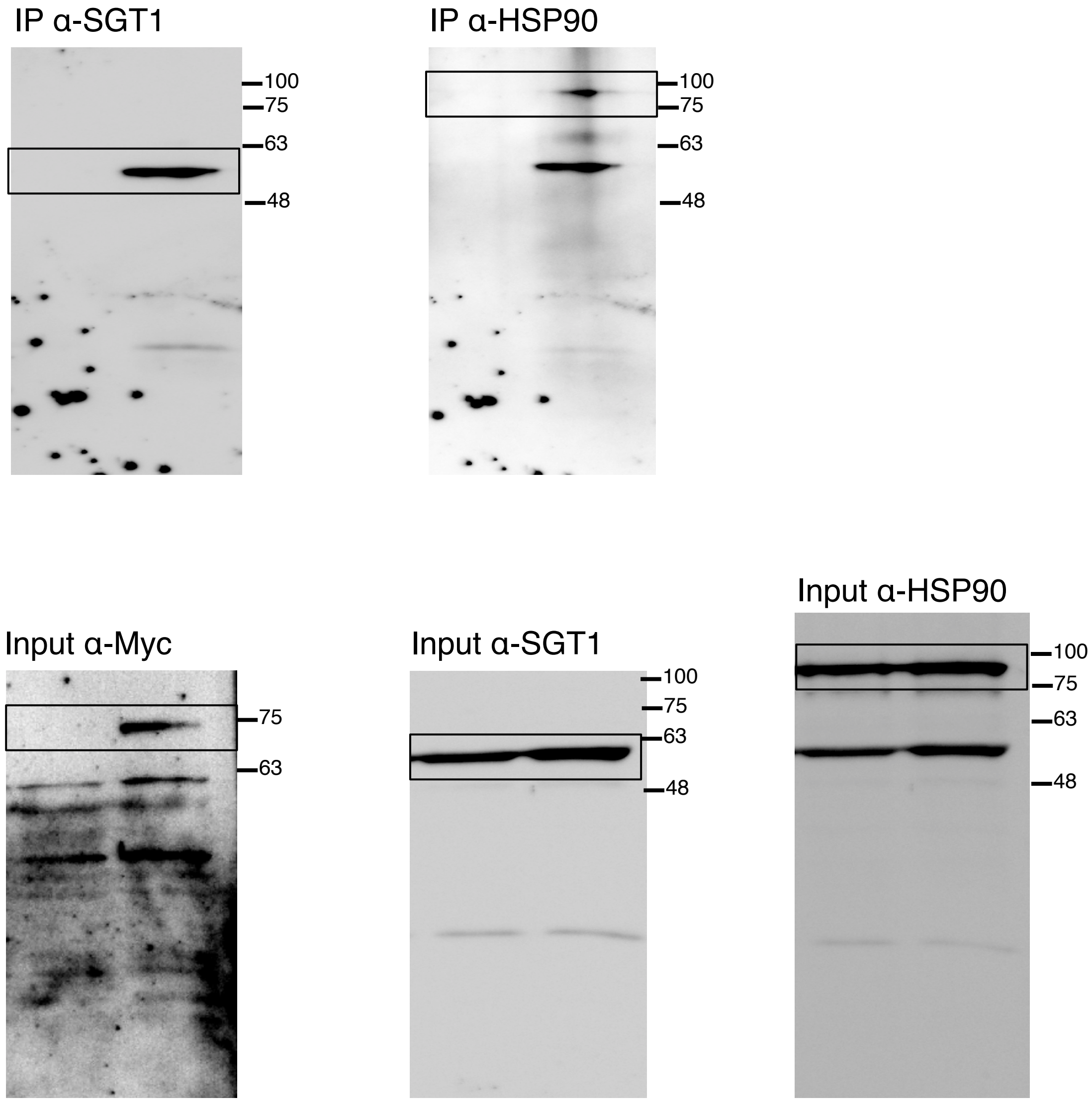
IP α-SGT1
100
75
63
48
IP α-HSP90
100
75
63
48
Input α-Myc
75
63
Input α-SGT1
100
75
63
48
Input α-HSP90
100
75
63
48

Figure 5b

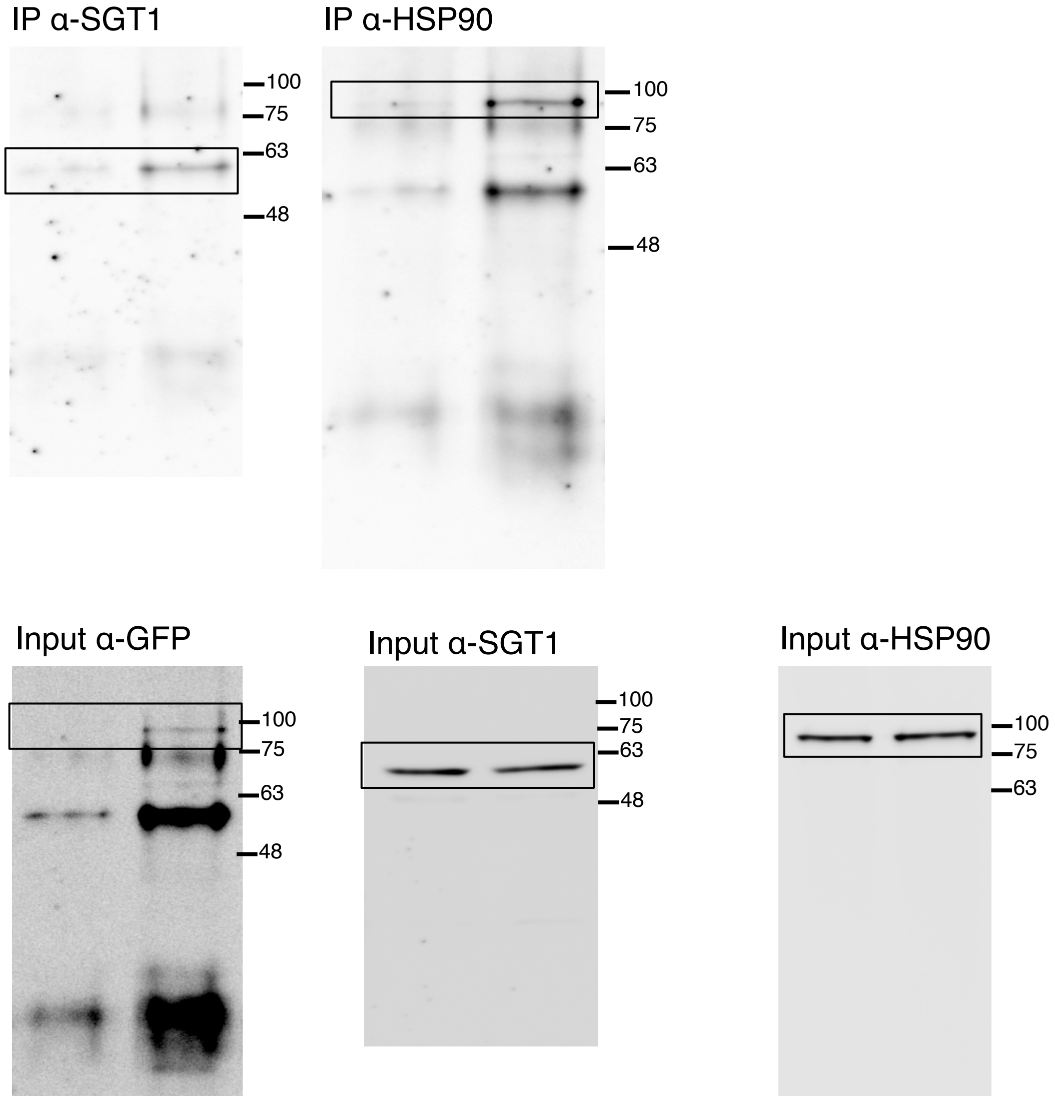

IP α-SGT1
100
75
63
48
IP α-HSP90
100
75
63
48
Input α-GFP
100
75
63
48
Input α-SGT1
100
75
63
48
Input α-HSP90
100
75
63

Figure 5c

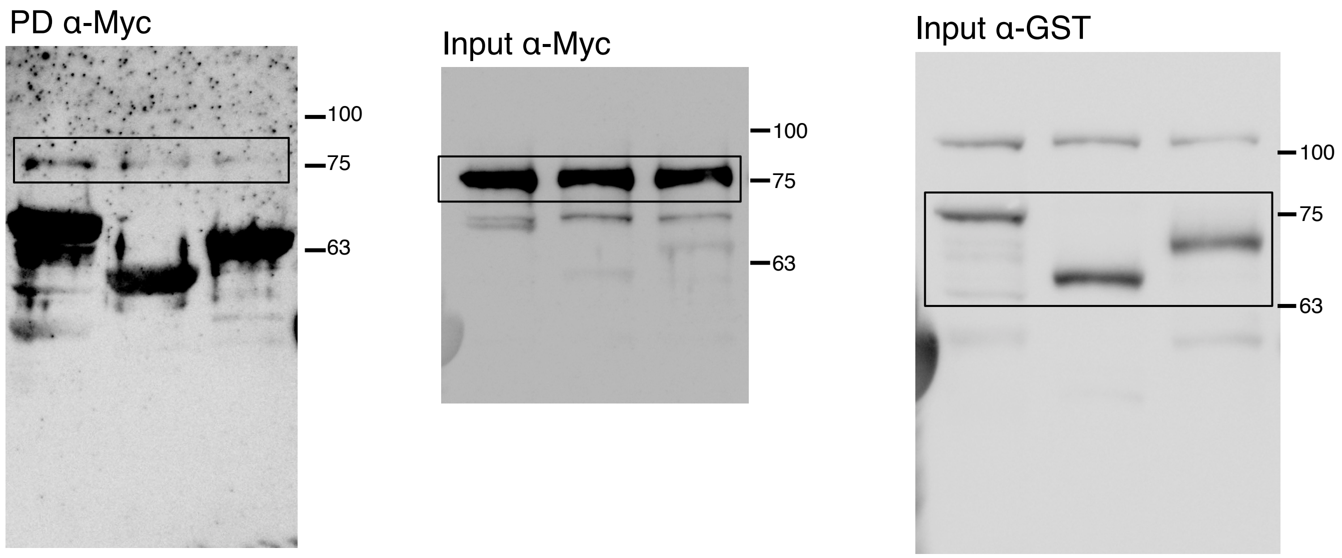

Figure 5d

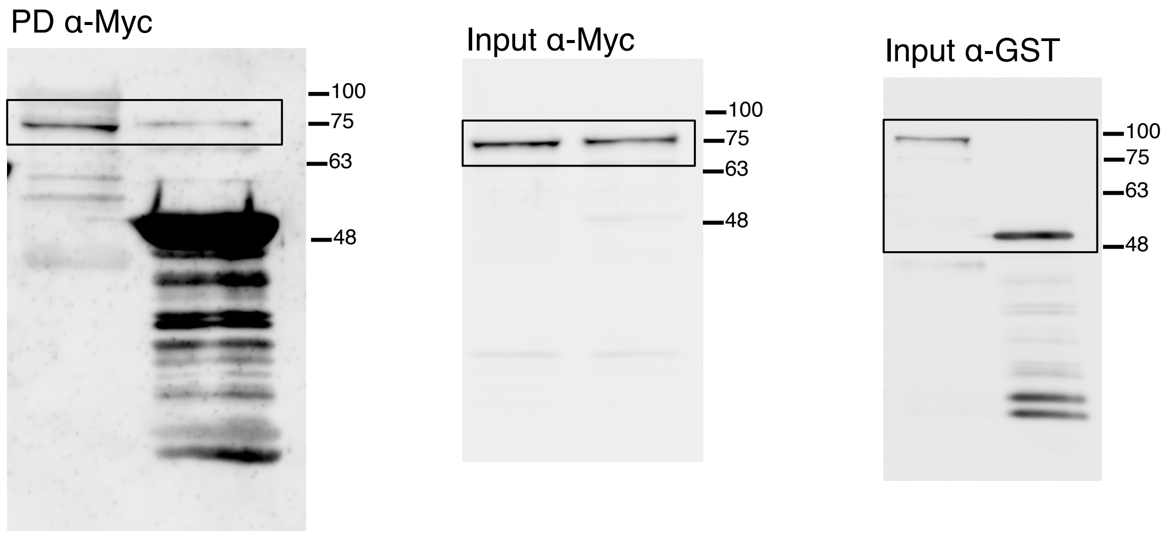
PD α-Myc
100
75
63
48
Input α-Myc
100
75
63
48
Input α-GST
100
75
63
48

Figure 5k

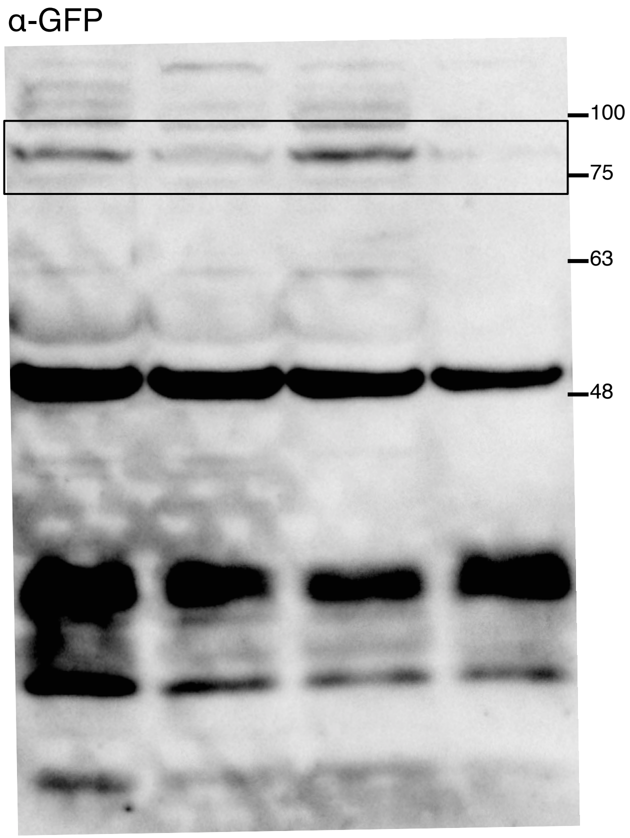

Blot for Suppl. Fig. 8

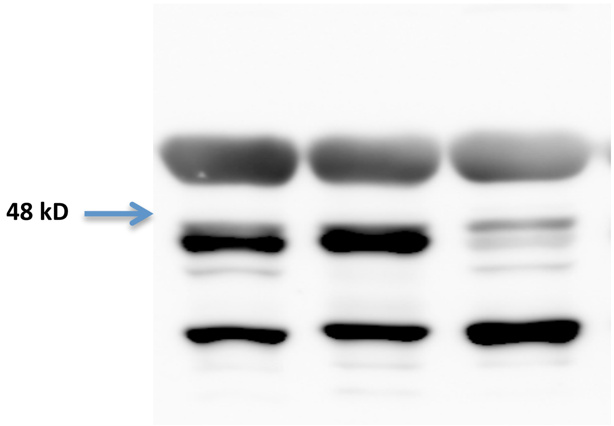

Supplementary Table 1: Primers used in this study

**TIR1/AFB-VENUS lines**	
TIR1	
EcoR1 pTIR1F	5'- TCAGAATTCTAATATGACCACGTCGGCAAACGCAAG-3'
TIR1 BamH1	5'- CTAGGATCCTAATCCGTTAGTAGTAATGATTTGCCTGGA-3'
BamH1 Venus	5'- CTAGGATCCGTGAGCAAGGGCGAGGAGCT-3'
Venus Stop Not1	5'- ATAGCGGCCGCTACTTGTACAGCTCGTCCATGCCGAGA-3'
Not1 3'TIR1	5'- TATGCGGCCGCGACAAAGAAACGAGGTACATTTTTCATTAATCC-3'
3'TIR1 Not1	5'- TTAGCGGCCGCGTGAGTTGTTTCTGATATTTGACTACTTGTGC-3'
AFB2	
Kpn1 pAFB2F	5'- ATCGGTACCGAATATTCCAACTATATGCTTATGGTTGAATCAC-3'
AFB2 EcoRV	5'- TCGGATATCGAGAATCCACACAAATGGCGGCGCAT-3'
EcoRV Venus	5'- ATCGATATCGTGAGCAAGGGCGAGGAGCT-3'
Venus Stop Not1	5'- ATAGCGGCCGCTACTTGTACAGCTCGTCCATGCCGAGA-3'
Not1 3'AFB2	5'- ATAGCGGCCGCGTCCTTTTCAGACCAAACTGGTTCTGAT-3'
3'AFB2 Not1	5'- TAAGCGGCCGCGGATGACTTGCCTGATCTTTTTCACCTC-3'
AFB3	
Kpn1 pAFB3F	5'- ATAGGTACCAAGCAGCTGAAGAGGTTGAGAGTGG-3'
AFB3 BamH1	5'- TCAGGATCCAAGAATCCTAACATATGGTGGTGCATCTTTTCTAGTCC-3'
BamH1 Venus	5'- CTAGGATCCGTGAGCAAGGGCGAGGAGCT-3'
Venus Stop Not1	5'- ATAGCGGCCGCTACTTGTACAGCTCGTCCATGCCGAGA-3'
Not1 3'AFB3	5'-TAGGCGGCCGCTCTCTTTGCACCTTCATGTGTTTTC-3'
3'AFB3 Not1	5'-TCAGCGGCCGCAGGTTCGTTCGTAATCACTTCTCC-3'
**Pull-down and BiFC assays**	
clTIR1_F	CACCATGCAGAAGCGAATAGCCTT
clTIR1_R	TTATAATCCGTTAGTAGTAATG
SGT1B_F	CACCATGGCCAAGGAATTAGCAGAGA
SGT1B_R	TCAATACTCCCACTTCTTGAGCT
SGT1B(-SGS)_R	TCACACTGGTCTCTGCGACAGCGCTGAT
HSP90.2CDS_F	AGATGGAGGAAGTCGACAAGGGTGGGCGCGCCGACCCAGCTT
HSP90.2CDS_R	AAGCTGGGTCGGCGCGCCCACCCTTGTCGACTTCCTCCATCT
IAA7_F	CACCATGATCGGCCAACTTATGAACCT
IAA7_R	TCAAGATCTGTTCTTGCAGTACT
**qRT-PCR**	
IAA5_F	CCGGAGAAAGAACAGTCTCG
IAA5_R	AGCATCCGAACAGAATTTGC
IAA14_F	GAAGCAGAGGAGGCAATGAG
IAA14_R	CCCATGGTAAAGGAGCTGAA
IAA19_F	GGTGACAACTGCGAATACGTTACCA
IAA19_R	CCCGGTAGCATCCGATCTTTTCA
TIR1_F	ATCGCTGCCACTTGCAGGAATC
TIR1_R	TGGCCACTAACGTCGTCAACATC
AFB2_F	GCCGCTAATTGCAGGCATCTTC
AFB2_R	AGTCGTGCAAGTGTCTGGGAAAC
AFB3_F	AGGTTGAAGCGGATGGTTGTAACAG
AFB3_R	GCAAGTCCAGCTCACGAAGATGC
GH3.5_F	CCATCTCTGAGTTCCTCACAAGC
GH3.5_R	TCCTCTTCGATTGTTGGCATTAGC
GH3.17_F	ACGCAGACACGTCATCAATCCC
GH3.17_R	TGCTGTGACGTGGCTTTAGCTC
VENUS__F	ACGTAAACGGCCACAAGTTC
VENUS__R	AAGTCGTGCTGCTTCATGTG
